# Compression systems for venous leg ulcers: a network meta-analysis and cost-effectiveness analysis

**DOI:** 10.1016/j.eclinm.2026.104065

**Published:** 2026-07-14

**Authors:** Han Phung, Catherine Arundel, Jo Dumville, Marta Soares, Pedro Rafael Saramago Goncalves, VenUS 6 investigators, Ross Atkinson, Una Adderley, Ian Chetter, Nicky Cullum, Tom Davill, Jane Griffiths, Catherine Hewitt, Charlotte Hirst, Katherine Jones, Maartje Kletter, Julie Mullings, Gareth Roberts, Brigid Smart, Philip Stather, Nikki Stubbs, Jude Watson, Sabeen Zahra

**Affiliations:** a Centre for Health Economics, University of York, York, YO10 5DD, UK; b York Trials Unit, Department of Health Sciences, Faculty of Science, University of York, York, YO10 5DD, UK; c Division of Nursing, Midwifery and Social Work, School of Health Sciences, Jean McFarlane Building, University of Manchester, Oxford Road, Manchester, M13 9PL, UK

**Keywords:** Venous leg ulcer, Compression system, Network meta-analysis, Economic evaluation

## Abstract

**Background:**

Whilst strong compression is effective in treating venous leg ulcers, compression systems vary in their effects on ulcer healing and wider outcomes. Four-layer bandage systems (4LB) and two-layer hosiery (2LH) are recognised effective compression systems in practice. The relative effectiveness and cost-effectiveness of two-layer bandage systems (2LB, a commonly used system), compression wraps (CW, a newer option), and short-stretch bandages (SSB) were uncertain. This paper aims to compare the effectiveness on time to venous ulcer healing and cost-effectiveness of 4LB and 2LH (or a choice of these), with 2LB, SSB, and CW.

**Methods:**

We updated the Ovid MEDLINE search of the Cochrane review on compression systems to identify new—up to 02 February 2026–relevant randomised controlled trials (RCTs), in addition to the individual patient data from VenUS 6—a recent large RCT comparing a choice arm of 4LB and 2LH, with 2LB and CW. We identified RCTs evaluating any type of compression systems (bandage or stockings). Study’s risk of bias was assessed using the Cochrane Collaboration tool. We used network meta-analyses to estimate relative treatment effects on time to ulcer healing, and used a Markov model to compare the cost-effectiveness of interventions over a lifetime horizon and from a UK NHS perspective. The primary outcome for the network meta-analysis was the hazard ratio (HR) of time to ulcer healing for alternative compression systems. For the cost-effectiveness analysis, total costs, total quality-adjusted life years (QALYs), and Incremental Cost-Effectiveness Ratios (ICERs) were estimated.

**Findings:**

Overall, 21 trials with 2934 participants were included, of which 5 (24%) and 4 (19%) studies had a moderate and a low risk of bias, respectively. Due to the sparsity of data, fixed-effects models were used. Heterogeneity, transitivity and consistency were explored by comparison of population characteristics in included studies, comparison of fixed- and random-effects models, and assessment of consistency matrix. The HRs of time to ulcer healing compared with 4LB/2LH, when including all studies, were 0.972 (95% CI 0.851–1.107) for SSB, 1.044 (95% CI 0.859–1.255) for 2LB, 0.927 (95% CI 0.745–1.136) for CW. The HRs compared with 4LB/2LH, when including only studies with low and moderate risk of bias, were 0.954 (95% CI 0.823–1.099) for SSB, 1.004 (95% CI 0.817–1.213) for 2LB, 0.901 (95% CI 0.719–1.110) for CW. The HRs compared with 4LB/2LH, when including only studies with low risk of bias, were 1.009 (95% CI 0.856–1.187) for SSB, 10.995 (95% CI 0.792–1.233) for 2LB, 0.872 (95% CI 0.695–1.085) for CW. The cost-effectiveness analysis (when using the NMA results including all studies) showed small differences in total costs and total QALYs across treatments. Compared with 4LB/2LH, the total costs for SSB, 2LB, and CW were higher, of £390 (95% CI: 41–985), £345 (95% CI: −102 to 1127), and £348 (95% CI: −133 to 1118), respectively. Total QALYs differences versus 4LB/2LH were −0.0015 (95% CI: −0.0110 to 0.0064) for SSB, 0.0022 (95% CI: −0.0085 to 0.0100) for 2LB, and −0.0044 (95% CI: −0.0207 to 0.0079) for CW. SSB and CW were dominated by 4LB/2LH. The ICER of 2LB versus 4LB/2LH was £159,614/QALY gain.

**Interpretation:**

These findings suggest potential small differences in the clinical effectiveness of 4LB/2LH, 2LB, SSB, and CW, but there is uncertainty in the estimates. In the UK, 4LB/2LH emerges as cost-effective.

**Funding:**

National Institute for Health and Care Research (NIHR) Health Technology Assessment Programme (Project Reference: 128625).

**Research in context**

**Evidence before this study**

Current first-line strong compression treatments for venous leg ulcers include four-layer bandage and two-layer hosiery. Two alternative compression systems are available in the UK NHS: two-layer bandage and compression wrap, though evidence on their clinical and cost-effectiveness are limited. A randomised controlled trial (VenUS 6) was conducted to compare the clinical effectiveness of four-layer bandage and two-layer hosiery (including a choice between these) with two-layer bandage and compression wrap. In this study, we extend the VenUS 6 findings to consider available evidence on strong compression systems (including short-stretch bandage, another commonly used system not evaluated in VenUS 6). We searched the Ovid MEDLINE from January 2012 and 02 February 2026, for papers published in English, using terms related to leg ulcers and compression systems, stockings, dressings, bandages, hosiery, wraps. The search identified 1367 records.

**Added value of this study**

This is the largest contemporary synthesis of evidence on compression therapy for venous leg ulcers, combining individual participant data and aggregate data in a network meta-analysis. We analysed 21 randomised controlled trials, including two with individual participant data, with a total of 2934 participants. We found that offering use of the four-layer bandage or two-layer hosiery (including a choice between these), two-layer bandage and short-stretch bandage had similar effectiveness on time to ulcer healing, and offering compression wraps had lower likelihood to be more effective than four-layer bandage or two-layer hosiery. An economic evaluation indicated that offering use of four-layer bandage or two-layer hosiery was expected to be cost-effective in the UK.

**Implications of all the available evidence**

Findings from this network meta-analysis support the continued use of the four-layer bandage or two-layer hosiery (including a choice between these) as first-line treatment for venous leg ulcer. Two-layer bandage and short-stretch bandage are comparably effective alternatives. Of all compression systems, four-layer bandage or two-layer hosiery (including a choice between these) offer better value for money in the UK.

## Introduction

Venous leg ulcer(s) are common open wounds on the lower leg resulting from venous hypertension caused by impaired blood flow in the leg veins. In England in 2015/16, there were an estimated 21,119 leg ulcers, with a healthcare cost burden of £102 million.^1^ In the United States, approximately 2.2% of Medicare beneficiaries suffer from leg ulcers, with an annual cost burden of $5.9 billion.^2^ A recommended first line treatment for venous leg ulcers is strong compression systems.^3^ Strong compression systems are elastic compression systems applying ≥40 mmHg at the ankle or a non-elastic (e.g., short stretch) systems applied in accordance with manufacturers’ recommendations. Compression provides graduated pressure from the ankle (highest pressure) to the knee to reduce intravenous pressure.

Whilst strong compression is effective, there are several options that may have different relative impacts on time to healing and wider outcomes. Four-layer bandage systems (4LB) and two-layer hosiery (2LH) are considered standard care^4^; their effectiveness on time to ulcer healing is largely comparable as evidenced by VenUS IV, a high-quality randomised controlled trial (RCT).^5^ Short-stretch bandages (SSB), although less common, are still used in UK practice.^6^ Another type of compression used to treat venous leg ulcers is two-layer bandage systems (2LB), which are currently commonly used in the UK NHS but with less evidence.^7^ Adjustable hook-and-loop-fastened compression (compression wrap, CW) is a newer treatment for this patient group. Both 2LB and CW lacked robust comparative evidence of their relative clinical- or cost-effectiveness.^8^, ^9^, ^10^, ^11^, ^12^

VenUS 6 (VENous Ulcer Study 6) was a clinical trial that evaluated the clinical effectiveness of 2LB and CW compared with evidence-based compression, ‘EBC’, a pragmatic arm allowing a choice between 2LH and 4LB using clinical judgement and patient preference.^13^^,^^14^ VenUS 6 found that the hazard of healing is reduced for those offered CW compared with EB, and that significant differences in healing rates between EBC and 2LB are unlikely.^14^

In this paper, we incorporate the findings of VenUS 6 with external evidence on all relevant comparator treatments using a network meta-analysis (NMA) and cost-effectiveness analysis to comprehensively assess the clinical- and cost-effectiveness of alternative compression systems. This aims to inform clinical and funding decisions about strong compression treatments for the treatment of venous leg ulcers.

## Methods

In this work, we assess the clinical and cost-effectiveness of strong compression for venous leg ulcers. The primary clinical outcome was time to ulcer healing. All relevant outcome data were identified through a systematic review of RCTs and quantitatively synthesised with individual patient data from VenUS 6 in an NMA. Cost-effectiveness evidence was modelled from the UK perspective and extrapolates the relative treatment effects (obtained from the clinical effectiveness review) to lifetime costs and health consequences. The NMA and cost-effectiveness analysis followed prespecified analysis plans (see Appendix 1 in Supplementary material).

### Evaluated compression systems

The strong compression systems evaluated include trials arms offering 4LB, 2LH, 2LB, CW, and SSB. Other forms of strong compression not commonly used in clinical practice but that have been evaluated in relevant RCTs (*ad hoc* systems) have been retained in the NMA when contributing to inferences but results for these have not been analysed. A full description of evaluated and *ad hoc* compression systems is available in Appendix 2 in Supplementary material.

### Systematic review

**Fig. 1:**
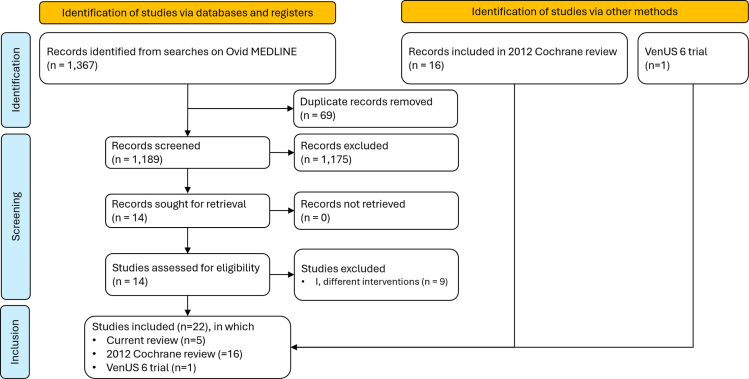
PRISMA flow diagram of study selection.

We updated an existing systematic review, by considering eligible studies published from 2012 onwards.^15^ The searches for the review update were first run in March 2022, re-run in July 2024 and on 02 February 2026, using Ovid MEDLINE library. We followed the same eligibility criteria as our previous review: briefly, we included RCTs comparing types of strong compression for the treatment of venous leg ulcers.^5^ Two researchers screened the references and made independent decisions about the inclusion or exclusion of records. Data was extracted by one researcher and checked by a second. We assessed each study risk of bias using the Cochrane Collaboration tool.^16^ Disagreements were resolved by discussion. We extracted data on treatments, duration of follow-up, number of participants, participants’ baseline characteristics (ulcer duration and ulcer size), and information on healing outcomes including the number of participants with healed ulcers. We had access to individual patient data for three eligible RCTs comprising a total of 1477 participants (VenUS 6,^13^ VenUS IV,^5^ and VenUS I^6^) on time to healing and baseline characteristics (e.g., age, gender, ulcer size and duration at baseline, study sites). Compression systems were classified by clinical experts using a classification system aligned with that used previously.^5^ The PRISMA diagram is presented in Fig. 1. Further details on the review are available in Appendix 3 in Supplementary material.

### Statistical analysis

To assess the comparative effectiveness of compression systems simultaneously, we applied an NMA using evidence from VenUS 6, VenUS IV, VenUS I and the RCTs identified in the systematic review. The NMA methodology follows the approach outlined in the VenUS IV NMA model.^17^

The classification of treatments in the primary analyses grouped 4LB, 2LH or a choice between these into a single treatment category. This group will be referred to thereafter as 4LB/2LH. This grouping was motivated by the specification of the EBC arm in VenUS 6, in which 4LB and 2LH were mixed. This specification was, itself, motivated by the evidence from VenUS IV, a large high-quality study that compared 2LH to 4LB and found no evidence of a difference in effectiveness. The grouping of EBC, 4LB and 2LH assumes that their effectiveness is equal. Also, because we here consider 4LB and 2LH together, data from VenUS IV could not be formally included in the NMA because this study compares the two treatments against each other. This grouping, however, allows the evidence from VenUS 6 on 2LB and CW against EBC to inform policy and practice (i.e., compare the effectiveness of the current first-line treatments 4LB and 2LH with 2LB and CW).

Our analysis synthesises relative effectiveness results on the hazard ratio scale. It assumes that the hazard of healing over time of venous leg ulcers follows a similar pattern (i.e., can be described using the same parametric time to event distribution) across treatments, and in both aggregated data and individual patient data. Commonly used parametric distributions are defined by two parameters: ancillary and location. The NMA model assumed that the ancillary parameter is common across studies and that treatments and estimated relative treatment effects (a hazard ratio) act to modify the location parameters. The parametric survival distribution that best fitted the individual patient time to healing data was selected using visual inspection and goodness-of-fit statistics (Akaike Information Criterion, AIC, and Bayesian Information Criterion, BIC).^18^^,^^19^ The proportional hazards assumption was evaluated using cumulative hazard plots and Schoenfeld residual tests.^20^ The performance of fixed- and random-effects models were compared using the total residual deviance and Deviance Information Criterion (DIC).^21^ Heterogeneity, transitivity, and consistency were assessed through comparison of fixed- and random-effects models, examination of the distribution of baseline severity, follow-up duration, and outcome definitions across treatment comparisons, and evaluation of a consistency matrix, respectively. Further supportive analyses to assess model assumptions are presented in Appendix 8 in Supplementary material.

The results from the NMA are presented as hazard ratios (HRs) for time to ulcer healing for each comparator compression system relative to the reference treatment, 4LB/2LH, along with 95% credible intervals (CrIs). A higher HR indicates faster time to ulcer healing. We also calculated treatment rankings based on the surface under the cumulative ranking curve (SUCRA).^22^ A higher SUCRA value indicates a higher likelihood of a treatment being ranked the best. The uncertainty surrounding the HR point estimates is shown using density strip plots. We conducted a sensitivity analysis including only studies judged at low or moderate risk of bias, and another including only low risk of bias studies. Sensitivity analyses using alternative survival distributions were also conducted.

Models were implemented under a Bayesian approach in WinBUGS, linked to the statistical software R.^23^^,^^24^ The MCMC sampler was run for two chains with alternative starting values, sampling 5000 ‘burn-in’ iterations and for further 5000 iterations per chain, on which inferences were based.^25^ Chain convergence and autocorrelation were assessed by inspecting density, history, and Gelman-Rubin graphic outputs for the different chains. Non-informative prior distributions were used to inform estimated parameters. More details on the methods for NMA models are available in Appendix 4 in Supplementary material.

### Cost-effectiveness analysis

**Fig. 2:**
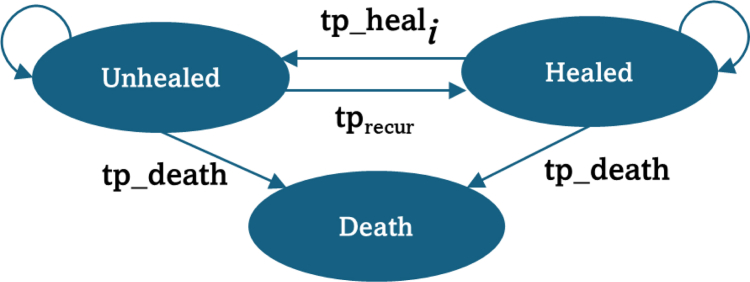
Analytic-decision model schematic. Abbreviations: tp_heal_i_ = transition probabilities from unhealed to healed health state for treatment *i*; tp_recur_ = transition probabilities of recurrence (shared for all treatments); tp_death = mortality risk.

This analysis considered the UK perspective and conformed to the National Institute for Health and Care Excellence reference case.^26^ It used 2024 costs, quantifies health outcomes as quality-adjusted life years (QALYs) and discounts future results to present value. We used a decision-analytic model, updating the one used in VenUS IV,^5^ to extrapolate evidence of the relative effectiveness of compression systems in the healing venous leg ulcers and to link to evidence on long-term health and cost outcomes. The model is a Markov state-transition model with three health key health states: unhealed ulcer, healed ulcer, and death (see Fig. 2). The model summarised and extrapolated time spent unhealed with different treatments not only considering time to healing of the first ulcer but also the possibility of recurrence. It quantified the impact on costs and health-related quality of life of having an unhealed ulcer over the individual’s lifetime. Evidence to inform the model, on time to recurrence, health-related quality of life, mortality, treatment costs and resource use were mainly sourced from VenUS 6, supplemented with data from VenUS I, VenUS IV, and the EVRA trial.^27^ These studies are high-quality, and specific for UK population. For 4LB/2LH, the economic analyses costed the offer of either treatment as per the EBC arm of the VenUS 6 trial. Model parameters are summarised in Table 1. A range of scenario analysis was conducted including using results from alternative NMA models, recurrence rates, and exceed mortality risk. Details on the methods of cost-effectiveness analysis and list of scenario analyses are available in Appendix 5 in Supplementary material. Details on the literature review for cost-effectiveness models and for economic model parameterisation are available in Appendix 6 and Appendix 7 in Supplementary material.

**Table 1:** Summary of economic model parameters.

Parameters	Model input	Data source
Population characteristics		
Reference ulcer duration (months)	10.0 months	VenUS 6
Reference ulcer area (cm^2^)	24.8 cm^2^	VenUS 6
Starting age (years)	70.3 years	VenUS 6
Transition probability (see Fig. 2)		
Transition probability from unhealed to healed (tp_heal_i_)	Modelled as time-to-event outcome, following a cumulative distribution function of a log-normal distribution, varying depending on treatments Log-normal regression coefficients: For 4LB/2LH: • μ: 1.543 (95% CrI 1.311–1.773) • β_log_ulcer_area_: 0.251 (95% CrI 0.198–0.305) • β_log_ulcer_duration:_ 0.082 (95% CrI 0.061–0.103) • Shape (γ): 0.896 (95% CrI 0.798–0.998) For other treatments: • HRs (on log scale) from NMA models are applied to tp_heal_i_ of 4LB/2LH	NMA model output
Transition probability of having a recurrent ulcer (tp_recur_)	Modelled as time-to-event outcome, following a cumulative distribution function of a log-normal distribution, the same for all treatments. Log-normal regression coefficients: • Scale: 8.077 (SE: 0.386) • Shape: 2.915 (SE: 0.293)	VenUS 6
Transition probability of dying (tp_death)	Age- and gender- related UK general population mortality, with a Standardised Mortality Risk of 1.79	UK lifetable^28^ SMR: VenUS 6
Treatment costs		
Unit prices (£) Mean (SE), £	4LB/2LH: • 4LB: 8.02 (0.66) • 2LH: 26.91 (1.47) 2LB: 9.03 (0.33) CW: 108.92 (6.65) SSB: 3.82 (0.27)	BNF^29^ Communication with study sites BNF^29^ Communication with study sites BNF^29^
Monthly costs Mean (SE), £	4LB/2LH: 18.05 (1.49) 2LB: 22.75 (1.60) CW: 31.58 (2.54) SSB: 15.09 (1.52)	VenUS 6
Resource use, treatment independent		
Monthly ulcer-related hospital outpatient visits Mean (SE)	0.04 (0.03)	VenUS 6
Monthly ulcer-related doctor consultations Mean (SE)	At GP practices: 0.03 (SE: 0.02) At home: 0.37 (SE: 0.54)	VenUS 6
Resource use, treatment dependent		
Duration of nurse visit Mean (SE), minutes	4LB/2LH: 31.03 (0.13) 2LB: 29.79 (0.22) CW: 31.31 (0.15) SSB: 32.89 (0.37)	VenUS 6
Monthly nurse visits Mean (SE)	4LB/2LH and SSB: 5.27 (0.05) 2LB: 5.44 (0.07) CW: 5.53 (0.05)	VenUS 6
Health state utility scores		
Healed	0.69 at baseline, adjusted for ageing over time using UK general population norms^30^	VenUS 6
Unhealed	Utility decrement of 0.087 (95% CI 0.055–0.119)	VenUS 6

2LB, Two-layer compression bandage systems; 4LB, Four-layer compression bandage system; 2LH, two-layer hosiery; BNF, the British National Formulary; CI, confidence interval; CrI, credible interval; CW, Compression wraps; EBC, Evidence-based compression; GP, general practitioner; NA, not applicable; SE, standard error; SSB, Short-stretch bandage.

Note: log-normal distributions were used to model uncertainty in costs and resource use, and beta distributions were used for utility data.

We present the lifetime costs and QALYs associated with each alternative treatment (discounted). To define whether a treatment is cost-effective, Incremental Cost-Effectiveness Ratios (ICERs) are calculated. An ICER is calculated by dividing the difference in costs between interventions (called the incremental cost) by the difference in QALYs between interventions (incremental QALYS). In the UK, and following NICE recommendations an ICER below the cost-effectiveness threshold of £20,000 indicates that the treatment is cost-effective.^26^ When multiple treatments are considered, as is the case with our analysis, the ICER for a particular intervention must be calculated against the next best intervention determined in a full incremental analysis. To avoid this complexity, we also present results in terms of net monetary benefit (NMB), defined as *NMB* = *Cost effectiveness threshold (here £20,000)* × *Total QALYs − Total Costs*, portraying the total monetary value of treatments after subtracting its associated costs. Using this metric, the treatment associated with the highest NMB is cost-effective. This leads to the same recommendation as the ICER.

To account for the uncertainty of parameters, the models were run probabilistic with 5000 Monte Carlo simulations using inputs randomly derived from the parameter distributions, except for the relative effects of alternative treatments, which were directly applied from the estimates extracted from the NMA model outputs. The probabilistic results are presented with 95% confidence interval (CI).

### Ethics

Ethical approval for the VenUS 6 study, of which the present study forms part, was obtained from the West of Scotland Research Ethics Committee 4 (reference 20-WS-0121). In the VenUS 6 trial, eligible patients were approached for written informed consent.

### Role of the funding source

This study was sponsored by the National Institute for Health and Care Research (NIHR) Health Technology Assessment Programme (Project Reference: 128625). The views expressed are those of the author(s) and not necessarily those of the NIHR or the Department of Health and Social Care. The Funder and Sponsor of the study had no role in study design, data collection, data analysis, data interpretation, writing of the report or the decision to submit the manuscript.

## Results

We included a total of 22 RCTs (Fig. 1): (i) five studies were identified from the 1367 records retrieved from the searches in this update, (ii) 16 studies were included in the VenUS IV NMA^11^^,^^12^^,^^31^, ^32^, ^33^; and (iii) VenUS 6 trial. Risk of bias assessment is presented in Appendix 3 in Supplementary material. The data used in the NMA (excluding VenUS IV) pertains to 21 RCTs: two providing individual patient data (VenUS I and VenUS 6),^6^^,^^13^ and 19 in aggregated data format (Table 2).^8^, ^9^, ^10^, ^11^, ^12^^,^^31^, ^32^, ^33^, ^34^, ^35^, ^36^, ^37^, ^38^, ^39^, ^40^, ^41^, ^42^, ^43^, ^44^

**Table 2:** Characteristics of randomised controlled trials included in the network meta-analysis.

ID	Study	Treatment	Follow up (weeks)	Number participants	Mean ulcer duration at baseline (months)	Mean ulcer size at baseline (cm^2^)	Number healed during follow-up	Risk of bias
Studies with individual patient data								
1	VenUS I^6^	4LB	52	195	3	3.81	107	Low
		SSB	52	192	3	3.82	86	
2	VenUS 6^13^	EBC	52	213	10.1	22.5	147	Low
		2LB	52	208	11.4	24.8	134	
		CW	52	212	8.4	27.3	131	
Studies with aggregated data								
3	Duby 1993^34^	4LB	12	25	20.5	11.9	11	High
		SSB	12	25	26.7	13.1	10	
4	Scriven 1998^35^	4LB	52	32	13	13.3	17.6	High
		SSB	52	32	21	8.3	18.24	
5	Partsch 2001^36^	4LB	16	53	1.25	1.5	33	High
		SSB	16	59	1	1.9	43	
6	Ukat 2003^37^	4LB	12	44	NA	17.7	13	High
		SSB	12	45	NA	12.2	10	
7	Franks 2004^38^	4LB	24	74	2	5	59	Moderate
		SSB	24	82	2	3.5	62	
8	Junger 2004b^39^	SSB	12	60	5.57	5.95	19	Moderate
		2LH	12	61	4.14	5.62	29	
9	Kralj 1996^40^	4LB	24	20	7.9	18.6	7	High
		Ba	24	20	6.9	17.2	8	
10	Polignano 2004b^41^	4LB	24	39	NA	10.1	29	High
		Zinc Paste	24	29	NA	9.3	19	
11	Wilkinson 1997^42^	4LB	12	17	NA	11.2	8	High
		BHeH	12	18	NA	8.6	8	
12	Colgan 1995^43^	4LB	12	10	9.3	27.5	6	High
		BzeaH	12	10	66.5	48.5	7	
13	Blecken 2005^10^	4LB	12	12	NA	50.08	4	High
		HV	12	12	NA	48.98	4	
14	Moffatt 2008^8^	4LB	4	42	48.8	5.7	3	High
		2LB	4	39	46.6	11.8	6	
15	Szewczyk 2010^9^	4LB	12	15	NA	6	9	High
		2LB	12	16	NA	5.3	10	
16	Wong 2012^44^	4LB	24	107	NA	NA	72	Low
		SSB	24	107	NA	NA	77	
17^a^	Harrison 2011^31^	4LB	52	215	11.7	3	178	Low
		SSB	52	209	11.4	3.3	192	
18^a^	Gillet 2019^32^	4LB	16	43	6.5	NA	10	High
		r2LB	16	49	9.4	NA	23	
19^a^	Mosti 2020^11^	CW	12	33	9	16	26	Moderate
		2LB	12	33	8	12.5	23	
20^a^	Stather 2021^12^	CW	26	20	NA	NA	12	Moderate
		Mixed SSB/2LB	26	20	NA	NA	11	
21^a^	Lazareth 2012^33^	4LB	12	93	6.81	10.29	78	Moderate
		2LB	12	94	6.42	9.75	82	

NA, not available; 4LB, Four-layer compression bandage systems; 2LH, Two-layer compression hosiery; EBC, Evidence-based compression; SSB, Short-stretch bandage; Paste, Zinc paste bandage; 2LB, Two-layer compression bandage systems; CW, Compression wraps; Ba, Adhesive bandage; BheH and BzeaH, The combination of bandage and hosiery with layers; mixed SSB/2LB, Choice of SSB and 2LB; r2LB, Reusable 2LB. For more details on classification system, see Ashby et al., 2014.^5^

a Randomised controlled trials identified in the updated systematic literature review on effectiveness of compression systems for venous leg ulcers.

**Fig. 3:**
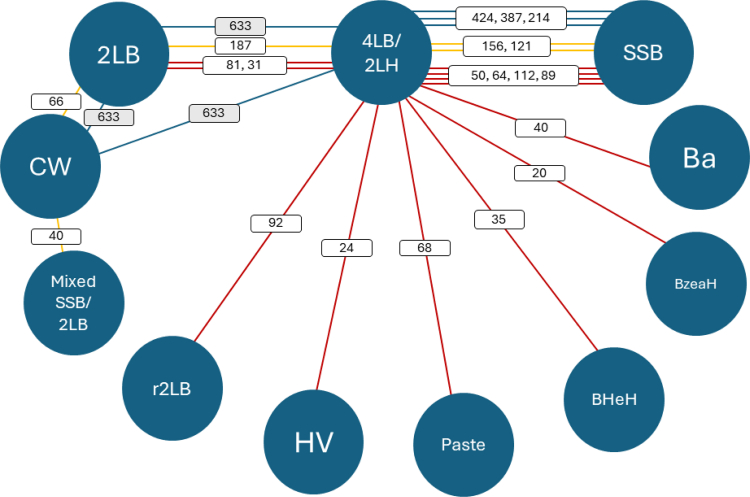
Evidence network of all studies. Number of participants per study indicated. Note: each line represents one study informing the pairwise comparison; the line colours represent the risk of bias for that study: red—high risk; yellow—moderate risk; blue—low risk of bias. The figures denote the number of participants in studies sharing the same risk of bias. Grey filled boxes denote a three-arm study (VenUS 6). Abbreviations: 4LB, Four-layer compression bandage systems; 2LH, Two-layer compression hosiery; SSB, Short-stretch bandage; Paste, Zinc paste bandage; 2LB, Two-layer compression bandage systems; CW, Compression wraps; Ba, Adhesive bandage; BheH and BzeaH, The combination of bandage and hosiery with layers; mixed SSB/2LB, Choice of SSB and 2LB; r2LB, Reusable 2LB. For more details on classification system, see Ashby et al., 2014.^5^

The evidence network of all studies is shown in Fig. 3. The included studies had a mix of low, moderate, and high risk of bias. We conducted three analyses considering the following structures:

1. Network 1: includes all studies (n = 21 studies);
2. Network 2: includes only studies with low and moderate risk of bias (n = 9 studies);
3. Network 3: includes only studies with low risk of bias (n = 4 studies).

The selection of time to event distributions to be used within the NMA model is detailed in Appendix 4 in Supplementary material. The log-normal survival distribution was selected for further use in the NMA model. The NMA models used in this section are fixed-effect models, given the limited number of treatment contrasts that contribute to the estimation of the between-study heterogeneity. The distributions of baseline severity, follow-up duration, and outcome definitions were broadly comparable across treatment comparisons, suggesting that the transitivity assumption was plausible. Examination of the consistency matrix (Appendix 9) revealed no major concerns, indicating a high level of consistency. Further supportive analyses assessing the underlying model assumptions indicated low-to-moderate between-study heterogeneity (τ^2^ = 0.11), no evidence of inconsistency within the network, and moderate confidence in the certainty of the evidence (detailed results are provided in Appendix 8 of the Supplementary material).

**Fig. 4:**
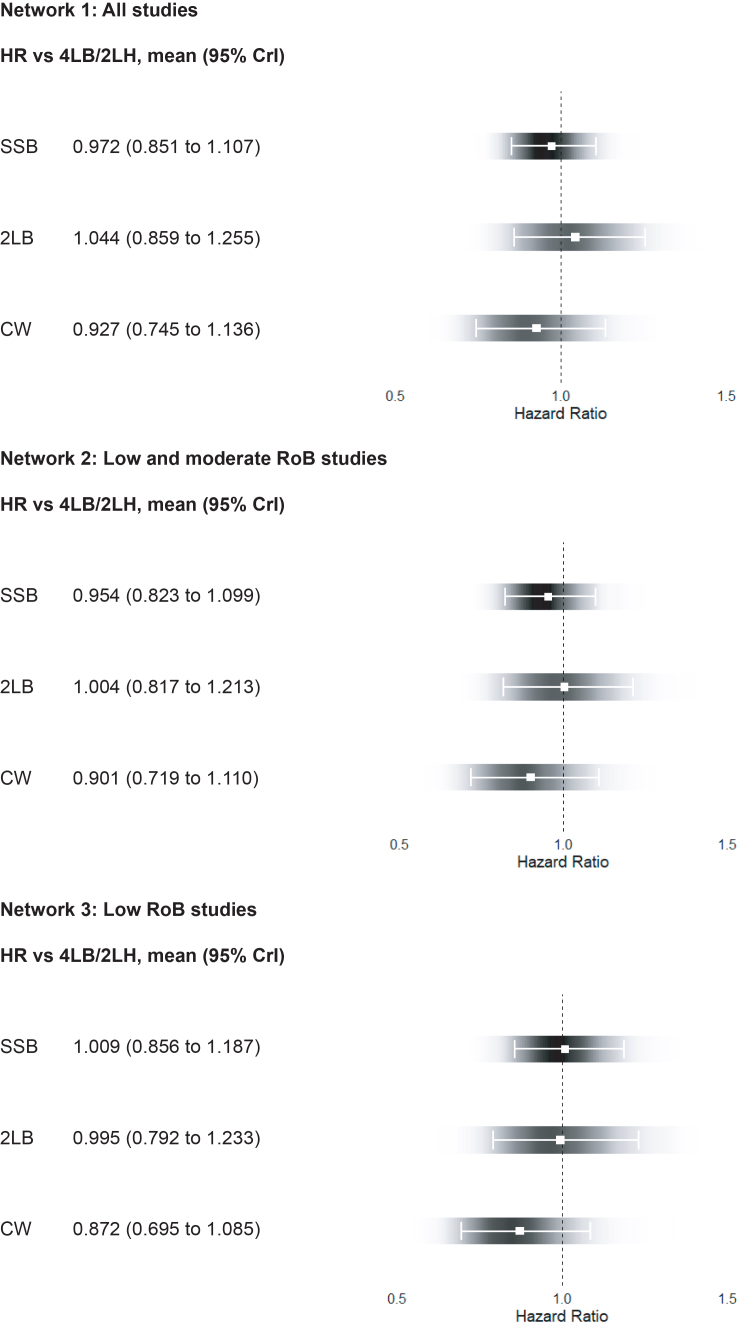
Network meta-analysis results (hazard ratio of time to ulcer healing and 95% credible intervals) sorted based on risk of bias of the included studies. Note: HR greater than 1 indicates reduced time to ulcer healing (better outcomes) against the reference treatment (2LH/4LB). The density strip plots are overlaid with mean HRs and their associated credible intervals both illustrating the uncertainty in the estimates. In the density strip plots, the colour gradient indicates the density of estimated HRs at specific values, with darker shading representing higher density (i.e., where HRs are more concentrated). The plots highlight regions of high or low concentration of the estimated HRs. Including these plots alongside Credible Intervals emphasise the distribution of estimated HRs from the NMA model, which are expected to be non-normally distributed. Plotting all HRs provides a clearer view of the spread of the estimated values. Abbreviations: 4LB, Four-layer compression bandage systems; 2LH, Two-layer compression hosiery; 2LB, Two-layer compression bandage systems; CrI, Credible Interval; CW, Compression wraps; EBC, Evidence-based compression; SSB, Short-stretch bandage.

Across all networks, there was no statistical significance in time to ulcer healing across compression systems. CW, albeit the differences did not reach statistical significance at 5% level, had the lowest HR point estimates compared with 4LB/2LH (Fig. 4). When studies with high and moderate risk of bias are progressively removed, the HRs for CW versus 4LB/2LH decrease (indicating a less favourable outcome for CW). The HRs for CW versus 4LB/2LH are 0.927 (95% CrI: 0.745–1.136) when all studies are included; 0.901 (95% CrI: 0.719–1.110) when studies with high risk of bias are excluded, and 0.872 (95% CrI: 0.695–1.085) when only studies with low risk of bias are included. The point estimate from network 3 is closer to the VenUS 6 results than those from networks 1 and 2, showing the added heterogeneity from studies with a higher risk of bias. The SUCRA rankings (Table 3) varied between networks: 2LB was the top-ranked treatment in network 1, 4LB/2LH in network 2, and 4LB/2LH and SSB in network 3. CW consistently ranked lowest in all networks.

**Table 3:** Network meta-analysis: Surface under the cumulative ranking curve, SUCRA.

SUCRA	Treatment			
	2LH/4LB	SSB	2LB	CW
Network 1: All studies	50%	44%	57%	35%
Network 2: Studies with low and moderate risk of bias	70%	49%	68%	31%
Network 3: Studies with low risk of bias	64%	64%	60%	12%

2LB, Two-layer compression bandage systems; CW, Compression wraps; EBC, Evidence-based compression; SSB, Short-stretch bandage; SUCRA, surface under the cumulative ranking curve, a higher score indicates a greater likelihood that a treatment is more effective than the others.

Results from a range of sensitivity analyses on different NMA models (random-effect versus fixed-effect, alternative assumptions on survival distribution of time to healing) remained consistent with the main findings. Results of these scenarios are available in Appendix 9 in Supplementary material.

The cost-effectiveness analysis (using NMA results from Network 1 for the base case) showed relatively small differences in total costs and total QALYs across all treatments (Table 4). Over a lifetime and compared with 4LB/2LH, compression treatments SSB, 2LB, and CW are associated with higher mean total costs of £390 (95% CI: 41–985), £345 (95% CI: −102 to 1127), and £348 (95% CI: −133 to 1118), respectively. Differences in mean total QALYs versus 4LB/2LH are also small, at −0.0015 (95% CI: −0.0110 to 0.0064) for SSB, 0.0022 (95% CI: −0.0085 to 0.0100) for 2LB, and −0.0044 (95% CI: −0.0207 to 0.0079) for CW. At a cost-effectiveness threshold of £20,000 per QALY (value typically used in UK NICE decision-making),^26^ our findings indicate that, in the UK, 4LB/2LH is considered a cost-effective treatment for the healing of venous leg ulcers. These cost-effectiveness findings remain consistent across a range of scenario analyses. Results of these scenarios are available in Appendix 10 in Supplementary material.

**Table 4:** The cost-effectiveness of compression systems.

Treatment	2LH/4LB (costed as EBC)	SSB	2LB	CW
Total costs £, mean (95% CI)	2634 (1099–5847)	3024 (1252–6418)	2979 (1253–6392)	2983 (1223–6495)
Total QALYs, mean (95% CI)	6.53 (6.16–6.89)	6.52 (6.16–6.89)	6.53 (6.17–6.89)	6.52 (6.16–6.88)
Incremental costs £, mean (95% CI)	Reference treatment	390 (41–985)	345 (−102 to 1127)	348 (−133 to 1118)
Incremental QALYs, mean (95% CI)	Reference treatment	−0.0015 (−0.011 to 0.0064)	0.0022 (−0.0085 to 0.01)	−0.0044 (−0.0207 to 0.0079)
ICER (£/QALY gained)	Reference treatment	Dominated^b^	159,614	Dominated^b^
NMB^a^, mean (95% CI)	127,870 (119,772–135,515)	127,450 (119,146–135,208)	127,568 (119,587–135,251)	127,434 (119,242–135,242)
Incremental NMB^c^, mean (95% CI)	Reference treatment	−419 (−1186 to 68)	−302 (−1221 to 347)	−436 (−1602 to 274)

2LB, Two-layer compression bandage systems; CI, confidence interval; CW, Compression wraps; EBC, Evidence-based compression; ICER, incremental cost-effectiveness ratio; NMB, net monetary benefits; QALYs, quality-adjusted life years; SSB, Short-stretch bandage.

a At a cost-effectiveness threshold of £20,000/QALY gained. A positive incremental net monetary benefit implies that the treatment is cost-effective.

b Dominated: the treatment is more expensive and offers less health benefits than the reference treatment.

c Incremental NMB indicates the differences in NMB of comparators against the reference treatment; positive values indicate that the treatment is cost-effective and negative values the opposite.

## Discussion

This study provides the most comprehensive and up-to-date evidence review and analyses of the relative clinical- and cost-effectiveness of strong compression treatments for active venous leg ulcers. The evidence base comprises 21 studies, including four large trials with low risk of bias (VenUS I, VenUS 6, Harrison 2011,^31^ Wong 2012^44^), alongside 17 smaller studies with moderate to high risk of bias. The VenUS trials contribute significantly to the NMA estimates, but heterogeneity is driven by the smaller, higher-risk studies which affect point estimates and increases overall uncertainty.

The network meta-analysis findings suggest no clear evidence of differences in time to ulcer healing between SSB, 2LB, and CW compared with 4LB/2LH. Analyses incorporating studies at different risks of bias find that the inclusion of studies at higher risk of bias (that were often also small) contributes to an overestimation of the effectiveness of comparators. However, across all analyses, 4LB/2LH and 2LB shared higher effectiveness rankings, indicating a higher probability of being clinically effective, whereas CW consistently ranked lowest. When, in the economic model, pooled relative treatment effects were extrapolated over a lifetime and integrated with treatment costs and resource use, 4LB/2LH was identified as the cost-effective option in the UK compared with alternative systems. The cost-effectiveness results were mainly driven by the treatment costs, given that differences in QALYs were small.

Our findings support the use of 4LB/2LH as a first-line compression therapy in the UK. Our findings also support the continued use of 2LB and SSB, which are commonly used within the UK NHS, as comparably effective alternatives. These findings are important for clinical practice, considering the ongoing innovation and adaptation of strong compression systems.

Previous studies, small and at high risk of bias, suggested that CW is an effective treatment for venous leg ulcers. In opposition, the VenUS 6 findings demonstrate the inferiority of CW compared with EBC. In our NMA, this difference did not quite reach commonly used levels of statistical significance. Differences here from the VenUS 6 findings can be explained by the inclusion of the small RCT on the model and differences in the assumptions of analyses used (a log-normal regression in the NMA and a Cox regression model in VenUS 6).^11^

With the evidence available at the time, the VenUS IV cost-effectiveness analyses identified 2LB as a cost-effective option. Our analyses revised these estimates to include larger and higher quality studies on the evidence base, including VenUS 6. Current results suggest that the 4LB/2LH group, when costed as evidence-based compression, i.e., the combination of 2LH and 4LB based on clinical judgement and patient preference as per the VenUS 6 study, is cost-effective in relation to 2LB. The ability to select compression systems—e.g., using bandaging in the early stages when the wound is leaking and bleeding, or for more severe cases, and hosiery for more mobile patients or those with controlled oedema—may therefore optimise treatment based on the individual and their ulcer and generate most efficient use of resources.^4^ Further research could examine the question of whether using 2LB as the bandaging option in EBC instead of 4LB could remain effective and cost-effective.

Our study has some limitations. First, as discussed, we were unable to examine hypothetical treatment strategies where 2LB or SSB might replace 4LB within EBC. Strategies that allow clinicians to choose the most appropriate option for different phases of ulceration may offer advantages in healing compared with a blanket policy. Second, there is considerable variation in how included RCTs in the NMA handled treatment switching, but we could not control for its impact on clinical effectiveness. Switching between compression systems is, however, common in practice; with the data being somewhat generalisable to clinical practice. We do, however, recognise that treatment changes could be more extensive outside the RCT context. Third, by grouping 4LB and 2LH into a single category of treatment in the NMA, we could not reassess the uncertainty surrounding the equivalence of the two compression systems given new bodies of evidence. Lastly, this study aimed to assess the effectiveness of strong compression systems–defined as delivering at least 40 mmHg at the ankle–however, the intended pressure in the included studies may not always have been achieved or maintained (although this strengthens the generalisability of this study’s results to clinical practice).

In conclusion, our findings show that 2LB, SSB and 4LB/2LH are expected to have similar effectiveness in the time to healing of venous leg ulcers. Point estimates suggest slightly slower healing for CW compared with 4LB/2LH but there is uncertainty around the estimates, and the findings are not statistically significant. For compression systems with clinical outcomes, clinical decisions should factor in individual circumstances, patient preference and, importantly, cost-effectiveness. We showed that, in the UK, offering a choice between 2LH and 4LB is likely to be cost-effective amongst the options considered.

## Contributors

JD, CA, PS, MS acquired the funding. JD, CA, PS, MS conceived and designed the study. CA, JD acquired the data. HP, PS, MS undertook the statistical analyses. PS supervised the analysis and writing of the manuscript. HP and PS drafted the manuscript, and this was revised with input from all writing committee members. All authors have read and approved the final manuscript. HP and PS accessed and verified the data. The VenUS 6 investigators oversaw the conduct of the study.

## Data sharing statement

Anonymised datasets generated and analysed during the current study will be stored in a publicly available open research repository (https://osf.io/echxv). Data will be made available via this repository following completion of analysis and subsequent publication. Sharing of this anonymised data is covered by original participant consent for VenUS 6 which permits sharing of data to support future research via sharing anonymously. Analysis code is available on https://github.com/HanPhung/VenUS6.

## Declaration of interests

In addition to other NIHR funding obtained by authors for studies outside of this work, authors have the following conflicts to declare: CA reports financial reimbursement from Society of Tissue Viability and Vascular Society of Great Britain and Ireland to facilitate meeting attendance to present the VenUS 6 study. All other named authors declare that they have no competing interests.

## Appendix A Supplementary data

Appendix 1–11

VenUS 6 investigators
