# Supplementary material for: Compression systems for venous leg ulcers: a network meta-analysis and cost-effectiveness analysis
Source: eClinicalMedicine. 2026 Jul 14;97:104065. doi: 10.1016/j.eclinm.2026.104065 (PMC13382440; doi:10.1016/j.eclinm.2026.104065)
Supplement: Appendix 1–11 [file mmc1.docx]

Supplementary Materials

*Title*: **Compression Systems for Venous Leg Ulcers: A Network Meta-Analysis and Cost-Effectiveness Analysis**

[Appendix 1. Link to Analysis Plans for Network Meta-Analysis and Cost-Effectiveness Analysis 2](#_Toc232796522)

[Appendix 2. Further Information on Compression Systems 2](#_Toc232796523)

[Appendix 3. Additional Detail on Systematic Literature Review for Treatment Effectiveness 3](#_Toc232796524)

[Appendix 4. Additional Detail on Statistical Analysis Supporting the NMA 4](#_Toc232796525)

[Appendix 5. Additional Detail on Methods for The Cost-Effectiveness Analysis 8](#_Toc232796526)

[Appendix 6. Additional Detail on the Literature Review for Cost-Effectiveness Decision Models 17](#_Toc232796527)

[Appendix 7. Additional Detail on the Targeted Literature Reviews for Economic Model Parameterisation 26](#_Toc232796528)

[Appendix 8. Supportive analyses for the assessment of inconsistency, heterogeneity, and certainty 28](#_Toc232796529)

[Appendix 9. Additional Results of Scenario Analysis for the NMA 32](#_Toc232796530)

[Appendix 10. Additional Results of Scenario Analyses for the Cost-Effectiveness Model 35](#_Toc232796531)

[Appendix 11. PRISMA and PRISMA-NMA checklists 39](#_Toc232796532)

[References 46](#_Toc232796533)

# Appendix 1. Link to Analysis Plans for Network Meta-Analysis and Cost-Effectiveness Analysis

Analyses plans have been deposited in the VenUS 6 trial ISRCTN page [ISRCTN67321719, <https://doi.org/10.1186/ISRCTN67321719>]. Direct links to the deposited documents are:

- Evidence synthesis analysis plan: <https://www.isrctn.com/editorial/retrieveFile/a1f0c5e9-6611-4163-96aa-dc3e5bed26d5/38740>
- Health economics analysis plan: <https://www.isrctn.com/editorial/retrieveFile/2aeaa1dc-04db-4770-a4fb-059dbceb8b3f/38740>

# Appendix 2. Further Information on Compression Systems

Descriptions of evaluated compression systems in this study are as follow:

1. 4LB, (an elastic system consisting of an orthopaedic wool layer plus three subsequent bandages);

2. 2LH, (smooth first layer, or understocking, providing light compression over which a second overstocking i.e. UK class II or III depending on the understocking slips on);

3. Short-stretch bandage, SSB (an inelastic bandage system where one to three rolls of bandage are applied over orthopaedic wool);

4. 2LB, (bottom layer with cohesive compression bandage - sub-compression wadding layer and cohesive bandage).

5. CW, (adjustable hook-and-loop-fastened compression).

In addition to policy relevant treatments, for completeness, the network meta-analysis (NMA) model also includes full compression treatments that are not commonly used in clinical practice (*ad hoc* treatments), including:

1. Adhesive bandage, Ba;

2. Hosiery with Velcro device, HV;

3. The combination of bandage and hosiery with layers: BheH;

4. The combination of bandage and hosiery with layers: BzeaH;

One randomised controlled trial (RCT), identified in the updated systematic literature review (SLR) on effectiveness, compared CW with a choice between SSB and 2LB.^1^ However, the information reported in this trial is insufficient to determine whether the effects of these two treatments (SSB and 2LB) can be assessed separately. Therefore, we have grouped these treatments together as a new compression practice:

5. Choice of SSB and 2LB, mixed SSB/2LB.

Another compression treatment identified in one of the relevant RCTs is named as reusable 2LB, ^2^ and is also included in the NMA model, although this treatment is not yet available in the UK:

6. Reusable 2LB, r2LB.

# Appendix 3. Additional Detail on Systematic Literature Review for Treatment Effectiveness

To identified RCTs evaluating the effectiveness of relevant high compression treatments, we conducted an update to the systematic literature review (SLR) in VenUS IV. In the update the searches were restricted to papers published from 2012 onwards. The searches were initially run in March 2022 and repeated in July 2024. The update of the SLR process was not registered and a separate protocol was not prepared. We used the search methods and selection criteria from the Cochrane review of compression for venous leg ulcers, and extracted relevant healing outcomes as reported in the Cochrane review. ^3^ Searches were run in Ovid MEDLINE library. Two researchers screened the references and made independent decisions about the inclusion or exclusion of records. Data was extracted by one researcher and checked by a second. Risk of bias assessment was also conducted by one reviewer and checked by another. Disagreements were resolved by discussion.

In March 2022 the search identified 1,060 citations, with 66 duplicates subsequently being removed. The remaining 994 records were screened, and 186 records were retrieved for full-text review. After full-text review, a total of 14 RCTs were considered relevant. All compression treatments within the 14 identified studies were classified by experts using a classification system aligned with that of VenUS IV. Since the decision problem considered only high compression treatments (>40 mmHg at the ankle), studies only evaluating treatments of lower levels of compression were excluded. Subsequently, only five of the 14 RCTs were found to consider relevant treatments for the VenUS 6 evidence synthesis. ^4^ ^2^ ^1^ ^5^ ^6^ The Cochrane risk-of-bias tool for randomized trials (RoB-2) was used to assess each study (see Table 1).^7^ In general, identified RCTs presented moderate to high risk of bias.

The search was re-run in July 2024, where 198 additional references were identified, since the previous search performed in March 2022. After removing duplicate entries, 195 records were screened. Thirteen records were deemed relevant and retrieved for full-text review. However, none of these records provided relevant information for the VenUS 6 evidence synthesis modelling. The PRISMA diagram of SLR is presented in .

The search was re-run on 2 February 2026, with results restricted to publications from 1 July 2024 onwards. A total of 109 additional records were identified and screened, none of which met the inclusion criteria. The PRISMA diagram of SLR is presented in Figure 1 in the main text.

Table 1 RoB-2 assessment of RCTs identified in the updated systematic review

|  | **Randomisation process** | **Deviations from intended interventions** | **Missing outcome data** | **Measurement of the outcome** | **Selection of the reported result** | **Overall bias** | **Comment for overall bias** |
| --- | --- | --- | --- | --- | --- | --- | --- |
| Harrison 2011 ^4^ | Low | Low | Low | Low | Low | Low | Unclear impact due to the difficulty in blinding |
| Gillet 2019 ^2^ | Some concerns | Some concerns | Low | High | Low | High | Outcome assessment methods were not presented |
| Mosti 2020 ^1^ | Some concerns | Some concerns | Low | Low | Low | Some concerns |  |
| Stather 2021 ^5^ | Low | Low | Low | Some concerns | Low | Some concerns | Unclear blinding in outcome assessment |
| Lazareth 2012 ^6^ | Low | Some concerns | Low | Low | Low | Some concerns |  |

# Appendix 4. Additional Detail on Statistical Analysis Supporting the NMA

Similarly to the VenUS IV synthesis model, the model brought together all available IPD and AD into the same synthesis model where both formats of evidence contributed to the estimation of treatment effectiveness (on time to ulcer healing), parameterized and synthesized as hazard ratio.^8^ To achieve this, we assumed that the hazard of healing of venous leg ulcers follows a similar pattern (i.e.,a common parametric distribution) for both AD and IPD and across treatments. It is, thus, necessary to examine the how the empirical covariate-adjusted hazard of healing observed in the available IPD compares. To do this, and assuming that the proportionality of hazards assumption is valid, we examined which survival parametric distribution (exponential, Gompertz, Weibull, log-normal, log-logistic) best fitted the Kaplan-Meier curves presented in each IPD. Visual inspection and goodness of fit (AIC and BIC statistics) were used to rank fitted distribution for each set of IPD.^9^ ^10^ The parametric distributions that consistently ranked higher across datasets, and which provided clinically valid long-term extrapolations, were considered the best fit.

**Results**

The proportional hazard assumption on time to healing was valid across treatments in VenUS 6, assessed by Schoenfeld residual tests (p = 0.1854 and p = 0.1923 for the comparisons of 2LB vs. EBC and CW vs. EBC, respectively). The cumulative hazard of time to healing for compression treatments (time on log scale) is presented in Figure 1. Proportional hazard assumptions were also valid for time to healing data in VenUS I and VenUS IV. ^11,12^

Figure 1 Cumulative hazard for time to healing of treatments in VenUS 6


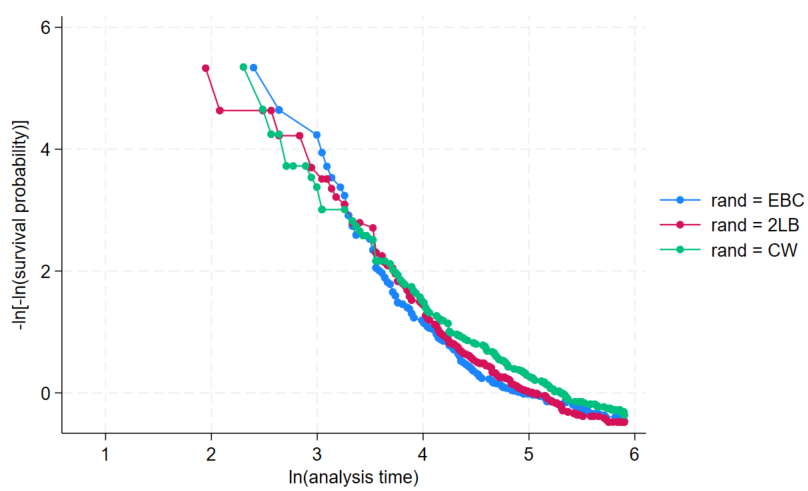


The visual assessment of distribution fitting is presented in Figure 2.

Figure 2 Visual fit assessment for alternative distributions on VenUS 6 time to healing data
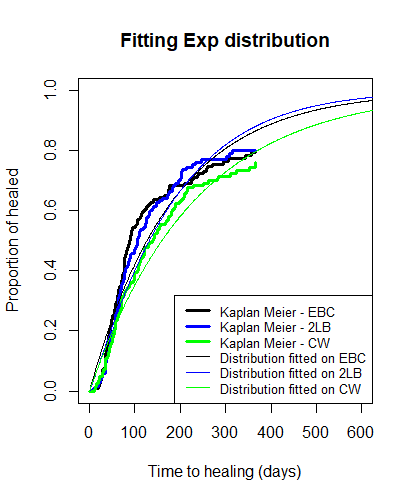

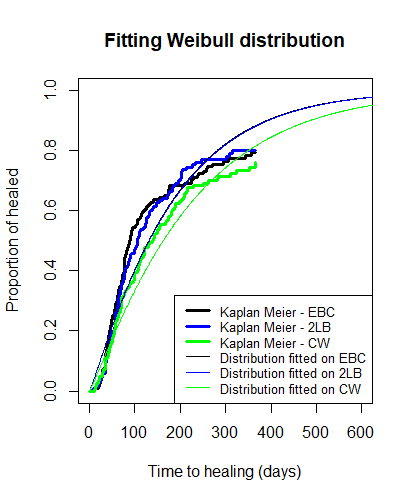

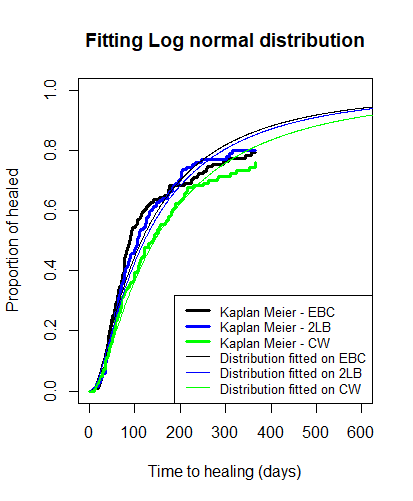

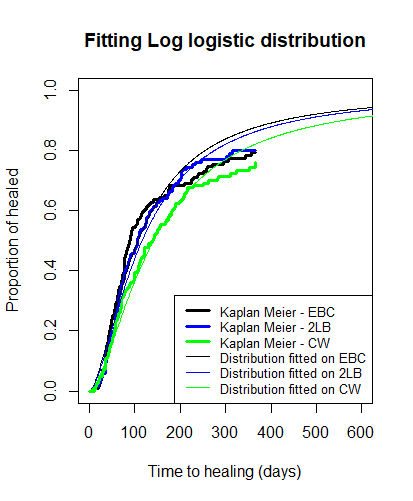

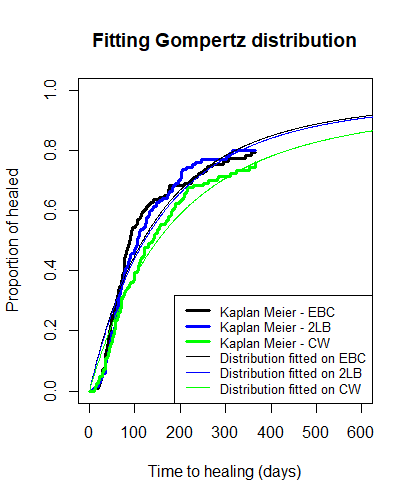


The above graphs indicate that models do not fit the data well, particularly post 200 days from randomisation. The Exponential, Weibull and Gompertz distributions substantially overestimate the EBC and 2LB time to healing estimates before 200 days. For that reason, the log-logistic and log-normal distributions visually appear to fit better to all three arms of the VenUS 6.

The statistical assessment via AIC and BIC suggests that the log-normal distribution provide the best fit (see Table 2) for VenUS 6 time to healing data, this was also the best fit for time to healing data in VenUS IV and VenUS I. ^8^

Table 2 Statistical fit assessment for alternative distributions on VenUS 6 time to healing data

|  | Adjusted (ulcer duration and size, centre effect) | | | |
| --- | --- | --- | --- | --- |
| Distribution | AIC | AIC rank | BIC | BIC rank |
| Weibull | 1,446 | 3 | 1,611 | 3 |
| Gompertz | 1,488 | 5 | 1,652 | 5 |
| Exponential | 1,487 | 4 | 1,647 | 4 |
| Log logistic | 1,361 | 2 | 1,526 | 2 |
| Log normal | 1,360 | 1 | 1,524 | 1 |

Abbreviations: AIC, Akaike Information Criterion; BIC, Bayesian Information Criterion

We thus selected Log Normal distribution for the NMA model.

The NMA model incorporates covariates including ulcer area and duration on natural logarithm scale, and centre effects. In scenario analyses we examined the robustness of outcomes according to alternative survival distribution assumptions. Thus, NMA models assuming a Log-logistic parametric distribution, which was the second best fit, and Weibull, which was used in VenUS IV NMA model, were implemented.

The model fit assessment suggests that both models fixed- and random-effects have similar performance in terms of total residual deviance whilst the Deviance Information Criterion in the random effects (RE) model was slightly lower than the fixed effects (FE). In the RE model, the estimation of the between-study heterogeneity parameter was based on 3 contrasts (out of a total of 11 contrasts) with more than one study informing them. These three contrasts contained moderate to high risk of bias studies and a strong assumption would need to be imposed to generalise the heterogeneity of the studies informing them to the rest of the network. Thus, the FE model was selected as the preferred approach.

Table 3 Model fit assessment

|  | Dbar | Dhat | pD | DIC | Total residual deviance |
| --- | --- | --- | --- | --- | --- |
| Fixed effect | 11954.7 | 11887.3 | 67.36 | 12022 | 304.7 |
| Random effect | 11941.9 | 11868.6 | 73.35 | 12015 | 304.6 |

The NMA models (WinBUGS and R) are available on https://github.com/HanPhung/VenUS6.

# Appendix 5. Additional Detail on Methods for The Cost-Effectiveness Analysis

## Analytic perspective

The economic analysis aims to determine which full compression treatment(s) for venous leg ulcers is cost-effective for use within the UK NHS. The study population considers people over the age of 18 years with at least one venous leg ulcer who are able and willing to tolerate full compression systems. The primary health outcome measure for this economic assessment was Quality-Adjusted Life Years (QALYs) derived from utility scores, obtained using the EQ-5D-5L health-related quality of life instrument which were then mapped to EQ-5D-3L. ^13^ The analysis takes the perspective of the UK NHS and Personal Social Services. ^14^ The time horizon includes the entire lifetime of the relevant population in compliance with current NICE guidance. ^14^ The health and cost outcomes are discounted at 3.5%; cycle length is one month. ^14^

## Decision-analytic model

The treatment effectiveness of various compression systems, measured as time to ulcer healing, is extrapolated over the lifetime health and cost outcomes using a decision-analytic model (DAM). This model builds on the DAM used in the VenUS IV study.^12^ A targeted literature review was conducted to ensure the model’s structure remains up-to-date and relevant; details of the review and its findings are provided in Appendix 6. Additional Detail on the Literature Review for Cost-Effectiveness Decision Models.

The model is a Markov state-transition model with three health key health states: unhealed ulcer, healed ulcer, and death (see Figure 1 in the main manuscript). Patients begin in the unhealed ulcer health state and receive one of alternative full compression systems. They either remain in the unhealed state or transition to the healed health state, with transition probabilities varying across treatments according to their effectiveness. Ulcers that heal may remain healed or recur, in which case patients transition back to the unhealed health state. The risk of ulcer recurrence is assumed to be independent of the previous compression treatment. The hazard of recurrence is modelled to depend on the time since ulcer healing by using 13 sub-health states with ‘healed’ status (e.g., healed-1, healed-2, etc.). In each sub-health state, patients remain ‘healed’ for only one month before either experiencing ulcer recurrence or dying. This division of health states is designed to track the time patients spent ‘healed’ and subsequently assign the corresponding transition probability of ulcer recurrence depending on this duration. For patients whose ulcers have not recurred after 12 months (i.e., those in healed-13), we assume they are free from ulcer recurrence thereafter. This is to align with findings from VenUS IV and VenUS 6 that the instantaneous risk of recurrence approaches zero after 12 months. Patients in any health state are at risk of death, which is assumed to be the same for both health states and is based on the general population mortality, with added mortality risk associated with venous leg ulcers.

We assessed the structural uncertainty of this model structure using a simpler version where the risk of ulcer recurrence is assumed to be independent of the time since ulcer healing (see Scenario analyses for further details).

## Model parameters

The transition probabilities from unhealed to healed health state for each full compression treatment are estimated through the NMA model. A series of targeted literature reviews were conducted to identify other potential sources for the model parameterisation (see Appendix 6. Additional Detail on the Literature Review for Cost-Effectiveness Decision Models for detail on the methods and results of these targeted literature reviews). However, none of the identified studies could provide higher quality evidence compared to VenUS 6, for which we have IPD. Therefore, model parameters including ulcer recurrence, health state utilities, mortality, resource use and costs were primarily based on data collected in the pivotal trial VenUS 6.

Data at the individual level was also made available from another trial - EVRA. ^15^ This trial assessed the effectiveness of early versus delayed endogenous treatment for patients with venous leg ulcers in the UK. While the surgical intervention in the treatment arm is not relevant to our study, the comparator arm, which reflects compression practices, is of relevance. Health-related quality of life data from EVRA was considered for analyses on utility.

**Time to ulcer healing**

The transition probabilities from unhealed to ‘healed 1’ health state are computed in two steps.

In the first step, the scale parameter (λ) specified for 4LB/2LH is estimated by using the mean coefficient values (reported in Table 4) and the regression coefficients resulted from the NMA

$\lambda_{4LB/2LH}= \mu+ \beta_{X} \times\bar{X}$,

where X represents covariates including ulcer size (log scale), ulcer duration (log scale), and a shared effect for study sites (β_new_centre_).

Table 4 Regression coefficients for the estimation of tp_heal_4LB/2LH_

| **Parameter** | **Mean** | **95% CrI** |
| --- | --- | --- |
| μ | 1.543 | 1.311 to 1.773 |
| β_log_ulcer_area_ | 0.251 | 0.198 to 0.305 |
| β_log_ulcer_duration_ | 0.082 | 0.061 to 0.103 |
| β_new_centre_ | 0.003 | -0.874 to 0.864 |
| Shape (γ) | 0.896 | 0.798 to 0.998 |

Abbreviations: CrI, credible interval

In the second step, the HRs of the remaining treatments compared to 4LB/2LH are applied to derive the transition probabilities for each treatment (HR of 4LB/2LH is set at 1):

$${p\_heal}_{i}\left( t \right)= 1-\Phi\left[ \frac{\log(t)-\lambda_{i}-log(\mathrm{HR}_{i})}{1/\gamma} \right]$$

$${p\_heal}_{i}\left( t-1 \right)=1- \Phi\left[ \frac{\log(t-1)-\lambda_{i}-log(\mathrm{HR}_{i})}{1/\gamma} \right]$$

$${tp\_heal}_{i}\left( t \right)= 1-\left[ \frac{{p\_heal}_{i}\left( t \right)}{{p\_heal}_{i}\left( t-1 \right)} \right]$$

where Φ is the cumulative distribution function for a standard normal distribution.

**Ulcer recurrence**

The transition probability from healed to unhealed health state was assumed to be the same for all compression treatments. This was assumed because no statistically significant differences were found across VenUS 6 treatments with respect to reference leg recurrence following complete healing of the reference ulcer. Within the economic model, ulcer recurrence was modelled using a log-normal distribution which was considered the best fit for the VenUS 6 trial data based on visual inspection and AIC/BIC ranking. Details on model fit assessment are presented next.

*Visual assessment*

Visual assessment on the smoothed hazard function of time to recurrence data (Figure 3) from VenUS 6 trial suggests distributions which can model non-monotonic hazard (e.g., log-logistic, log-normal, generalised gamma) may better fit to the data than distributions of monotonic hazard.

Figure 3 Smoothed hazard function of recurrence data VenUS 6


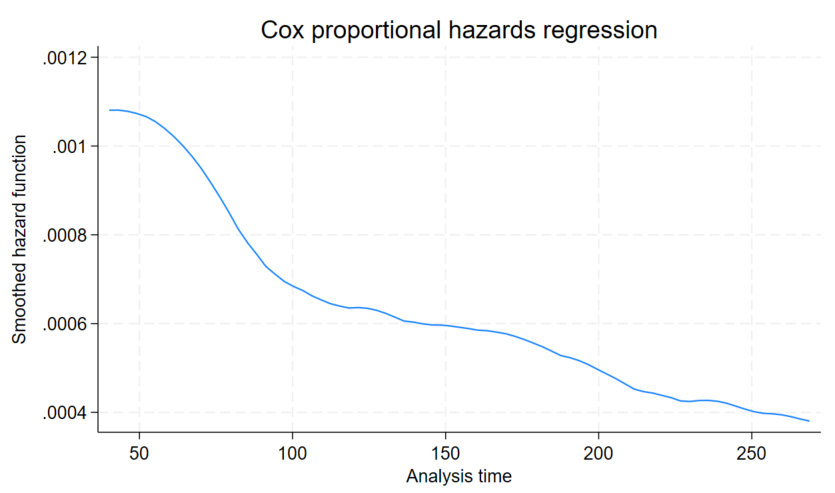


*Statistical assessment*

The AIC and BIC ranking (Table 5) suggests that the log-normal and the Generalised gamma distributions provide similar good fitting. The log-normal distribution was selected for the economic analysis. Log-normal distribution was also one of the best-fitting distributions for recurrence data in the VenUS IV trial. ^12^

Table 5 Model fit assessment – time to recurrence – VenUS 6 trial data

|  | AIC | rank AIC | BIC | rank BIC |
| --- | --- | --- | --- | --- |
| Weibull | 568 | 5 | 579 | 5 |
| Gompertz | 567 | 4 | 579 | 4 |
| Exponential | 585 | 6 | 593 | 6 |
| Log-logistic | 567 | 3 | 578 | 3 |
| Log-normal | 562 | 2 | 574 | 2 |
| Generalised gamma | 560 | 1 | 576 | 1 |

Abbreviations: AIC, Akaike Information Criterion; BIC, Bayesian Information Criterion

*Modelling of recurrence*

The results of the regression with log-normal distribution:

- Intercept λ: 8.077 (SE: 0.386)
- Shape γ: 2.915 (SE: 0.293)

The probability of recurrence at time *t* (with *t* $\leq$12 months) is defined as:

$${tp}_{recur}\left( t\leq12 \right)= \Phi\left[ \frac{\log(t)-\lambda}{\gamma} \right]$$

where Φ is the cumulative distribution function for a standard normal distribution. After 12 months, it is assumed that ${tp}_{recur}\left( t>12 \right)=0$.

Figure 4 Extrapolation of log-normal distribution fitted on time to recurrence data - VenUS6

Time (days)

*Scenario analyses*

In a scenario analysis, we assessed the potential structural uncertainty of the base case model by assuming a simpler structure that does not track the time patients stayed in “healed” state (i.e., the model considers only three health states: healed, unhealed and death, without splitting “healed” into 13 sub “healed” states). In this scenario, the risk of recurrence was, thus, assumed independent of the time since ulcer complete healing. The hazard rate of recurrence from VenUS 6 was applied within the economic model using a log-normal distribution.

In another scenario analysis, the ulcer recurrence rate was assumed constant over time with no recurrences after year 2 (i.e., from cycle 24^th^ onwards). The ulcer recurrence rate was derived from VenUS 6 data. In VenUS 6, 88 out of 464 patients (who healed) experienced ulcer recurrence over the follow-up period of 12 months, which is equivalent to an annual rate of 17.95%. This estimate is consistent with the recurrence rate observed in VenUS IV (18.95%).

**Health-related quality-of-life**

The VenUS 6 collected participant’s health-related quality of life data using the EQ-5D-5L at baseline, 1 month, 3 months, 6 months, and 12 months. The EQ-5D-5L scores were mapped to EQ-5D-3L using the Hernandez et al., 2022 algorithm ^13^ and using *eq5dmap* command in STATA. ^16^ A summary of EQ-5D-3L scores in VenUS 6 is provided in Table 6.

Table 6 EQ-5D-3L scores in VenUS 6 by ulcer healing

| Healing status | Baseline | | 1 month | | 3 months | | 6 months | | 12 months | |
| --- | --- | --- | --- | --- | --- | --- | --- | --- | --- | --- |
|  | N | Mean (SD) | N | Mean (SD) | N | Mean (SD) | N | Mean (SD) | N | Mean (SD) |
| Unhealed | 627 | 0.6 (0.29) | 432 | 0.62 (0.29) | 272 | 0.61 (0.29) | 178 | 0.59 (0.3) | 110 | 0.57 (0.31) |
| Healed | 0 |  | 59 | 0.66 (0.31) | 202 | 0.7 (0.29) | 258 | 0.7 (0.27) | 186 | 0.68 (0.29) |
| Unknown | 1 | 0.83 | 14 | 0.63 (0.34) | 4 | 0.68 (0.47) | 3 | 0.8 (0.12) | 0 |  |
| Missing | 6 |  | 129 |  | 156 |  | 195 |  | 338 |  |

Abbreviations: N, sample size; SD, standard deviation

The distribution of EQ-5D-3L index scores is left-skewed. To estimate the utility decrement from being in the unhealed health state, a simple linear transformation was applied to derive an average disutility score, i.e. 1- EQ-5D index scores at each timepoint. The rate of the utility decrement was observed not to fluctuate over time and between measurements. A Generalized Estimating Equations (GEE) model was used to estimate the effect of healing status:

$$\mathbb{E[}\left( 1-{EQ5D}_{it} \right)|{Heal}_{it}]=g({Heal}_{it}\beta)$$

where *i* denotes individual and *t* denotes the data collection points; *g(.)* denotes the link function; *heal* is a binary variable (1 if reference ulcer healed; 0 if unhealed). Quasi Information Criterion (QIC) was used to select the best correlation structure and the best fitting GEE model. ^17^ Identity link function g(.) with independent correlation structure and inverse Gaussian family distribution appeared to be the best fit. The GEE regression results are provided in Table 7.

Table 7 GEE model results (disutility values)

|  | Coefficient | SE | z | P>z | 95% CI |
| --- | --- | --- | --- | --- | --- |
| Healed | -0.087 | 0.016 | -5.35 | 0.000 | -0.119 to -0.055 |
| β | 0.397 | 0.011 | 34.98 | 0.000 | 0.374 to 0.418 |

Abbreviations: CI, confidence interval; SE, standard error

Unhealed ulcer is associated with a decrement in utility of 0.087 (95% CI 0.055 to 0.119). This decrement was selected for use in the base case economic model analysis.

The utility for patients in the healed health state was estimated by using the constant from GEE results (=1-0.397+0.087=0.69) and adjusted for aging over time using UK general population norms.^18^

An analysis was made to consider all relevant and available evidence on patients HRQoL to derive the utility decrement. A targeted review to identify relevant studies that informed the utility decrement between people with healed and unhealed ulcers was conducted (see Appendix 7. Additional Detail on the Targeted Literature Reviews for Economic Model Parameterisation), however, no additional study was found relevant, other than studies that we already had health related quality of life data (i.e., VULCAN, ^19^ VenUS I, VenUS IV, and VenUS 6). A fixed-effect meta-analysis was used to pool baseline-adjusted utility decrements at 3 months post-randomisation across these trials. The pooled value for the estimated utility decrement associated with unhealed ulcers was 0.0875 (SE: 0.012755).

Table 8 Fixed-effect meta-analysis of utility decrement associated with unhealed ulcers

| **Study** | **Mean adjusted utility decrement at 3 months (SE)** |
| --- | --- |
| **VULCAN** **^19^** | 0.003 (0.046) |
| **VenUS I** **^11^** | 0.110 (0.029) |
| **VenUS IV** **^12^** | 0.089 (0.045) |
| **VenUS 6** | 0.087 (0.017) |
| **Pooled value** | **0.0875 (0.013)** |

Abbreviations: SE, standard error

Another analysis performed considered the utility decrement at 6 weeks post randomisation for patients in the reference arm of the EVRA trial (mean: 0.1356, SE: 0.04) was also included in the meta-analysis. The mean utility decrement pooled across VULCAN, VenUS I, VenUS IV, VenUS 6, and EVRA was 0.0919 (SE: 0**.**01217).

The estimates from these two analyses were considered consistent with those from the regression analysis on repeated measurements in the VenUS 6 trial data; therefore, we do not present the economic results based on utility decrements from these two scenarios.

**Mortality**

The mortality risk of people with unhealed and healed ulcers were assumed to be the same (no evidence of a difference was found in a targeted review, details are provided in Appendix 7. Additional Detail on the Targeted Literature Reviews for Economic Model Parameterisation) and based on the age- and gender- related UK general population mortality.^20^ Mortality was adjusted to account for the increased risk of death associated with venous leg ulcers, via a Standardised Mortality Risk estimated from VenUS 6. In VenUS 6, 17 deaths were observed among 633 patients, equivalently a yearly morality rate of 2.69%. The general population mortality rate for 2023 for a similar population (age of 70.25 and 55% males) was 1.5%. Standardised mortality ratio of population of interest versus general population was thus estimated to be 1.79.

**Costs of compression systems**

Unit costs for compression treatments, including 4LB, 2LB, and SSB were sourced from the British National Formulary. ^21^ Unit costs for 2LH and CW were provided by VenUS 6 study sites.

Monthly treatment costs were sourced from VenUS 6 nurse visit reports. Treatment costs were calculated by dividing the total cost of all compression kits provided to participants while their ulcers were unhealed by the time to healing. Since CW and 2LH systems may be used as preventive treatments, it was possible that the last CW and 2LH provided were used beyond ulcer time to healing. To exclude the costs associated with prevention, we assumed that the final CW and 2LH issued were used for their respective lifespans (6 months for CW and 3 months for 2LH, as informed by our nurse specialists), with any costs incurred after healing were omitted. To estimate the cost of SSB, which was not a randomised treatment in VenUS 6, we assumed that the usage of SSB was the same as 4LB, however of different unit cost.

**Resource use**

The model considered only ulcer-related health resource use, meaning that no costs were incurred in the healed health state. Resource use in the unhealed health state encompassed nurse consultation visits (treatment dependent), doctor consultations, hospital visits and hospital stay related to ulcers (treatment independent). Unit costs associated for the different health resource use were sourced from the NHS reference costs database ^22^ and the Personal and Social Services Research Unit database. ^23^ Unit costs of nurse bands are obtained from the Personal and Social Services Research Unit database. ^23^

Table 9 Nurse resource use per month (dependent of treatment) sourced from VenUS 6

| **Compression**  **treatment** | **Number of nurse visits/month** | | **Duration of nurse visit (minutes)** | | **Average of nurse band** |
| --- | --- | --- | --- | --- | --- |
|  | Mean | SE | Mean | SE |  |
| **CW** | 5.53 | 0.05 | 31.31 | 0.15 | 5.05 |
| **4LB** | 6.12 | 0.07 | 32.02 | 0.15 | 5.33 |
| **2LH** | 4.45 | 0.05 | 29.79 | 0.22 | 5.21 |
| **EBC** | 5.27 | 0.05 | 31.03 | 0.13 | 5.28 |
| **2LB** | 5.44 | 0.07 | 37.04 | 0.20 | 5.33 |
| **SSB** | Assumed the same as 4LB | | 32.89 | 0.37 | 5.14 |

Table 10 Doctor visits, hospital daycare visits and hospital stay per month (independent of treatments) sourced from VenUS 6

|  | **Average number of visit/ month (SE)** | **Unit cost £** | **Sources** |
| --- | --- | --- | --- |
| **Doctor visit - Home** | 0.37 (0.54) | 95 | Assuming 5 miles and 12 minutes per travel; unit cost per mile travelled was £0.56 (PSSRU 2023) ^23^ and unit cost per 10 minutes was £42 (PSSRU 2023) ^23^ |
| **Doctor visit - GP** | 0.03 (0.02) | 42 | PSSRU 2023 ^23^ |
| **Doctor visit - Outpatient** | 0.25 (0.37) | 170 | NHS National Cost Collection 2023-2024 ^22^ |
| **Hospital visit (daycare)** | 0.04 (0.03) | 460 | Weighted average of day-case HRG data and outpatient procedures, NHS National Cost Collection 2023-2024 ^22^ |
| **Hospital stays** | 0.01 (0.04) | 601 | Non-elective inpatient short stay – Minor skin procedures, NHS National Cost Collection 2023-2024 ^22^ |

Abbreviations: GP, general practitioners; HRG, Health Resource Group; NHS, National Health Services, PSSRU, Personal Social Services Research Unit; SE, standard error

## Analyses

**Base case analysis**

The economic model estimates the lifetime costs and QALYs associated with each alternative treatment. To account for the uncertainty of parameters, the model is run probabilistic with 5,000 Monte Carlo simulations using inputs randomly derived from the parameter distributions, except for the relative effects of alternative treatments, which are directly applied from the Convergence Diagnostic and Output Analysis (CODA) extracted from the NMA model outcomes. The probabilistic results are presented with 95% confidence interval. Model was run in R 4.2.2 (the model is available in <https://github.com/HanPhung/VenUS6>).

**Scenario analysis**

The robustness of the findings in the base case analysis is tested by a range of scenario analyses (Table 11).

Table 11 List of scenario analyses to assess the robustness of the cost-effectiveness results

| **Items** | **Base case** | **Scenario** |
| --- | --- | --- |
| **Fixed effect vs random effect NMA model** | Fixed effect | Network meta-analysis considering a random effect with predictive distributions |
| **Alternative survival distributions for time to healing** | Log Normal | Log logistic |
| **Alternative survival distributions for time to healing** | Log Normal | Weibull |
| **Alternative NMA evidence networks** | Network 1 | Network 2 |
| **Alternative NMA evidence networks** | Network 1 | Network 3 |
| **Model structure** | Recurrence rate depends on time patients spent healed | Fixed recurrence rate within the first two years |
| **Model structure** | Recurrence rate depends on time patients spent healed | Recurrence rate is time dependent, but not dependent of time spent healed |
| **Exceed mortality rate** | Sourced from VenUS 6 (1.79) | The same as VenUS IV (2.36) |

Abbreviation: NMA, network meta-analysis

# Appendix 6. Additional Detail on the Literature Review for Cost-Effectiveness Decision Models

We conducted a structured literature review to identify recent (i.e., since 2011) decision-analytic models for venous leg ulcer and to help ensure whether the VenUS 6 model structure remains contemporaneous and relevant.

### Search strategies

To update the searches from the previous study (VenUS IV), a precision maximising search strategy was developed in Ovid MEDLINE. The strategy consisted of terms for leg ulcers combined with a narrow economic search filter to identify economic evaluations or economic models.^24^ Ovid MEDLINE, Ovid Embase and the NHS Economic Evaluations Database (NHS EED) were searched on 8^th^ August 2024 with a further search of CINAHL (via Ebsco) on 12^th^ August 2024. A date limit of 2011 onwards was applied and retrieval was restricted to English language studies. The search strategies can be found in the next section.

**Ovid MEDLINE(R) ALL**

1946 to August 07, 2024

Date of search: 8^th^ August 2024

Records retrieved: 161

1 *economics/ (10816)

2 exp *"costs and cost analysis"/ (81879)

3 (economic adj2 model*).mp. (15728)

4 (cost minimi* or cost-utilit* or health utilit* or economic evaluation* or economic review* or cost outcome* or cost analys?s or economic analys?s or budget* impact analys?s).ti,ab,kf,kw. (46188)

5 (cost-effective* or pharmacoeconomic* or pharmaco-economic* or cost-benefit or costs).ti,kf,kw. (93838)

6 (life year or life years or qaly* or cost-benefit analys?s or cost-effectiveness analys?s).ab,kf,kw. (43413)

7 (cost or economic*).ti,kf,kw. and (costs or cost-effectiveness or markov or monte carlo or model or modeling or modelling).ab. (92399)

8 1 or 2 or 3 or 4 or 5 or 6 or 7 (235809)

9 Leg Ulcer/ (8999)

10 Varicose Ulcer/ (5478)

11 (varicose ulcer$ or venous ulcer$ or leg ulcer$ or stasis ulcer$ or lower extremit$ ulcer$ or crural ulcer$ or ulcus cruris or ulcer$ cruris).ti,ab. (10963)

12 9 or 10 or 11 (16807)

13 8 and 12 (285)

14 markov chains/ (16344)

15 Stochastic Processes/ (16226)

16 Models, Theoretical/ (164273)

17 models, statistical/ (100642)

18 models, economic/ (11270)

19 monte carlo method/ (33139)

20 ((stochastic or mathematical or statistical or theoretical or population or process or probabili$ or simulat$ or monte carlo or markov) adj model$).ti,ab. (166637)

21 (stochastic process$ or markov process$ or markov chain$).ti,ab. (13643)

22 ((pharmacoeconomic$ or pharmaco-economic$ or decision$ or cost$) adj model$).ti,ab. (4942)

23 14 or 15 or 16 or 17 or 18 or 19 or 20 or 21 or 22 (450398)

24 12 and 23 (91)

25 13 or 24 (326)

26 limit 25 to yr="2011 -Current" (174)

27 (2011$ or 2012$ or 2013$ or 2014$ or 2015$ or 2016$ or 2017$ or 2018$ or 2019$ or 2020$ or 2021$ or 2022$ or 2023$ or 2024$).dt. (17163679)

28 25 and 27 (175)

29 26 or 28 (176)

30 exp animals/ not humans/ (5246522)

31 29 not 30 (176)

32 letter.pt. (1265988)

33 editorial.pt. (701078)

34 historical article.pt. (371093)

35 32 or 33 or 34 (2315505)

36 31 not 35 (168)

37 limit 36 to english language (161)

**Ovid Embase**

1974 to 2024 August 07

Date of search: 8^th^ August 2024

Records retrieved: 270

1 *economics/ (28000)

2 exp *"costs and cost analysis"/ (94106)

3 (economic adj2 model*).mp. (10737)

4 (cost minimi* or cost-utilit* or health utilit* or economic evaluation* or economic review* or cost outcome* or cost analys?s or economic analys?s or budget* impact analys?s).ti,ab,kf,kw. (70577)

5 (cost-effective* or pharmacoeconomic* or pharmaco-economic* or cost-benefit or costs).ti,kf,kw. (137615)

6 (life year or life years or qaly* or cost-benefit analys?s or cost-effectiveness analys?s).ab,kf,kw. (66526)

7 (cost or economic*).ti,kf,kw. and (costs or cost-effectiveness or markov or monte carlo or model or modeling or modelling).ab. (137147)

8 1 or 2 or 3 or 4 or 5 or 6 or 7 (334701)

9 leg ulcer/ (15520)

10 leg varicosis/ (1881)

11 (varicose ulcer$ or venous ulcer$ or leg ulcer$ or stasis ulcer$ or lower extremit$ ulcer$ or crural ulcer$ or ulcus cruris or ulcer$ cruris).ti,ab. (13914)

12 9 or 10 or 11 (21935)

13 8 and 12 (423)

14 exp markov chain/ (18064)

15 stochastic model/ (23250)

16 theoretical model/ (92783)

17 statistical model/ (178621)

18 economic model/ (3675)

19 exp monte carlo method/ (56324)

20 ((stochastic or mathematical or statistical or theoretical or population or process or probabili$ or simulat$ or monte carlo or markov) adj model$).ti,ab. (194164)

21 (stochastic process$ or markov process$ or markov chain$).ti,ab. (13582)

22 ((pharmacoeconomic$ or pharmaco-economic$ or decision$ or cost$) adj model$).ti,ab. (7223)

23 14 or 15 or 16 or 17 or 18 or 19 or 20 or 21 or 22 (513565)

24 12 and 23 (139)

25 13 or 24 (497)

26 limit 25 to yr="2011 -Current" (292)

27 letter.pt. (1336796)

28 editorial.pt. (818573)

29 note.pt. (998198)

30 27 or 28 or 29 (3153567)

31 26 not 30 (279)

32 limit 31 to english language (270)

**NHS Economic Evaluations Database (NHS EED)**

https://www.crd.york.ac.uk/CRDWeb/

Inception to date of closure - 31^st^ March 2015

Date of search: 8^th^ August 2024

Records retrieved: 16

1 MeSH DESCRIPTOR leg ulcer IN NHSEED 33

2 MeSH DESCRIPTOR Varicose Ulcer IN NHSEED 23

3 ((varicose or venous or leg or stasis or lower extremit* or crural) adj ulcer*) IN NHSEED 63

4 ((ulcus or ulcer*) adj cruris) IN NHSEED 0

5 #1 OR #2 OR #3 OR #4 63

6 (*) IN NHSEED FROM 2011 TO 2015 6121

7 #5 AND #6 16

**CINAHL** via Ebsco

Inception to 12^th^ August 2024

Date of search: 12^th^ August 2024

Records retrieved: 270

S1 (MH "Leg Ulcer") OR (MH "Venous Ulcer") 6,797

S2 TI (varicose or venous or leg or stasis or lower extremit* or crural) N0 ulcer*) OR AB (varicose or venous or leg or stasis or lower extremit* or crural) N0 ulcer*) OR TI (ulcus or ulcer*) N0 cruris) OR AB (ulcus or ulcer*) N0 cruris) 5,306

S3 S1 OR S2 7,968

S4 (MH "Costs and Cost Analysis") 19,599

S5 (MH "Cost Benefit Analysis") 38,357

S6 (MH "Cost Effectiveness Analysis") 1,481

S7 TI ( cost* N2 (effective* OR utilit* OR benefit* OR minimi* OR analy* OR outcome OR outcomes) ) OR AB ( cost* N2 (effective* OR utilit* OR benefit* OR minimi* OR analy* OR outcome OR outcomes) ) 70,321

S8 TI economic N0 evaluation* OR AB economic N0 evaluation* 6,763

S9 TI ( costeffective* OR costutilit* OR costbenefit* OR costminimi* ) OR AB ( costeffective* OR costutilit* OR costbenefit* OR costminimi* ) 305

S10 S4 OR S5 OR S6 OR S7 OR S8 OR S9 103,886

S11 S3 AND S10 378

S12 ( TI (cost* or economic*) OR AB (cost* or economic*) ) AND ( TI (markov or monte carlo or model or modeling or modelling) OR AB (markov or monte carlo or model or modeling or modelling) ) 51,895

S13 S3 AND S12 101

S14 (MH "Models, Theoretical") 52,449

S15 (MH "Models, Statistical") 40,215

S16 (MH "Decision Trees") 3,186

S17 TI ( (stochastic or mathematical or economic or statistical or theoretical or population or process or probabili* or simulat* or monte carlo or markov) N0 model*) ) OR AB ( (stochastic or mathematical or economic or statistical or theoretical or population or process or probabili* or simulat* or monte carlo or markov) N0 model*) ) 25,678

S18 S14 OR S15 OR S16 OR S17 112,448

S19 S3 AND S18 78

S20 S11 OR S13 OR S19 Limiters - Publication Date: 20110101-; English Language 257

S21 EM 2011- 4,751,554

S22 S11 or S13 or S19 460

S23 S21 AND S22 Limiters - English Language 259

S24 S20 OR S23 270

### Results of the structured literature review for the cost-effectiveness models

A total of 447 results returned from MEDLINE, Embase and NHSEED, with 298 after duplicates removed. An additional of 270 records were retrieved from CINAHL and leaving 171 results after removing deduplicates. In total, 469 records were included for title and abstract screening, from which 54 articles were retrieved for full text review. After excluding 22 articles that lacked sufficient details or did not specify a decision model, 32 studies were included for extraction (see Figure 5). The main characteristics of eligible studies were summarised in Table 12.

Figure 5 PRISMA diagram of literature review for cost-effectiveness models


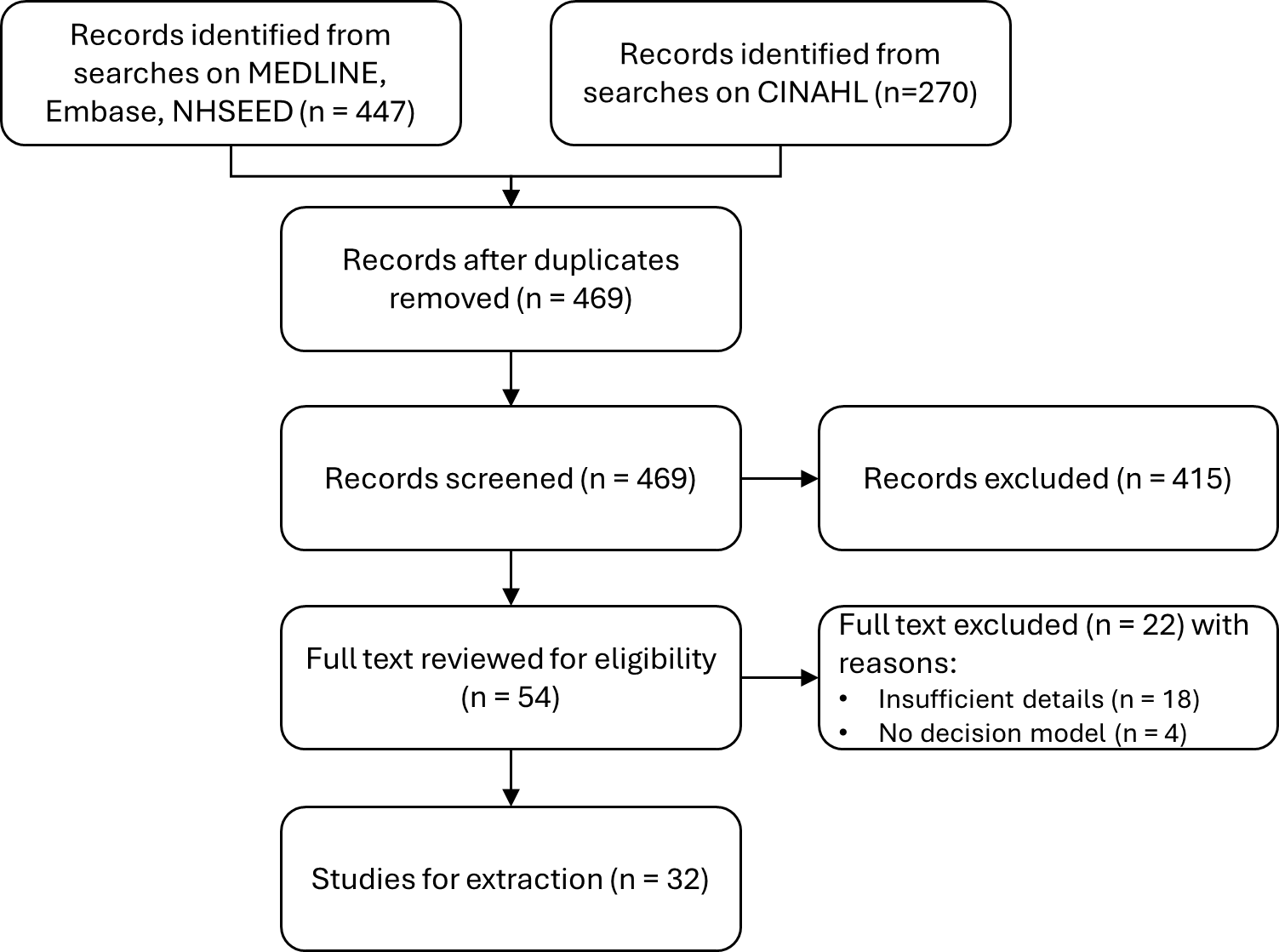


Table 12 Main characteristics of eligible studies reporting decision models for venous leg ulceration/ diabetic foot ulceration

| **#** | **Study** | **Title** | **Population** | **Treatment** | **Time horizon** | **Model design** | **Key health states** | **Perspective** | **Outcomes** |
| --- | --- | --- | --- | --- | --- | --- | --- | --- | --- |
| 1 | Brain 2019 ^25^ | Cost-effectiveness analysis of an innovative model of care for chronic wounds patients | Chronic wounds patients | Specialist wound care clinic, usual care | 1 year | Markov | Uncomplicated, healed, complicated with hospitalisation, dead | Societal perspective in Australian setting | Costs, QALYs |
| 2 | Taylor 2011 ^26^ | Modelling the cost-effectiveness of electric stimulation therapy in non-healing venous leg ulcers | Patients with VLUs | Electric stimulation therapy | 5 months | Markov | Improved, unchanged, worsened, healed, recurred ulcer | UK NHS and PSS | Costs, QALYs |
| 3 | Panca 2013 ^27^ | Clinical and cost-effectiveness of absorbent dressings in the treatment of fullly exuding VLUs | Patients with fullly exuding VLUs | Absorbent dressings | 6 months | Decision tree | Unchanged ulcer, improved ulcer, worsened ulcer, healed ulcer and death | UK NHS and PSS | Costs, QALYs |
| 4 | Jemec 2014 ^28^ | Cost-effective use of silver dressings for the treatment of hard-to-heal chronic venous leg ulcers | Hard-to-Heal Chronic VLUs | Silver Dressing | 4 weeks | Decision tree | No improvement, healing ulcer, healed ulcer | UK NHS and PSS | Costs, QALYs |
| 5 | Ashby 2014 ^12^ | VenUS IV (Venous leg Ulcer Study IV) - compression hosiery compared with compression bandaging in the treatment of venous leg ulcers: a randomised controlled trial, mixed-treatment comparison and decision-analytic model | Patients with VLUs | Full pressure compression | Lifetime | Markov | Healed, unhealed, death | UK NHS and PSS | Costs, QALYs |
| 6 | Carter 2014 ^29^ | Cost-effectiveness of three adjunct cellular/tissue-derived products used in the management of chronic venous leg ulcers | People with chronic venous leg ulcers | Three topically applied cellular/tissue-derived products used as adjunct therapies to standard care | 1 year | Markov | Unhealed, healed, and death | Payer’s perspective | Cost per ulcer-free week |
| 7 | Romanelli 2016 ^30^ | Difficult-to-heal wounds of mixed arterial/venous and venous aetiology: a cost-effectiveness analysis of extracellular matrix | Mixed arterial/ venous (A/V) or venous leg ulcers (VLUs) | Extracellular matrix, standard of care | 32 weeks | Markov | Unhealed, healed | Payer's perspective | Cost per closed wound |
| 8 | Augustin 2016 ^31^ | Cost-effectiveness of treating vascular leg ulcers with UrgoStart( R) and UrgoCell( R) Contact | People with vascular leg ulcers | Hydroactive dressing containing a nano-oligosaccharide factor (NOSF), neutral foam dressing without NOSF. | 8 weeks | Decision tree | Success (>40% reduction of wound area), failure | German statutory health care | Cost per responder |
| 9 | Nherera 2016 ^32^ | Estimating the Clinical Outcomes and Cost Differences Between Standard Care With and Without Cadexomer Iodine in the Management of Chronic Venous Leg Ulcers Using a Markov Model | Patients with VLUs | Standard Care With and Without Cadexomer Iodine | 1 year | Markov | Unhealed, healed, infected, death | US payers | Costs, QALYs, ulcer-free weeks |
| 10 | Guest 2017 ^33^ | Cost-effectiveness of using adjunctive porcine small intestine submucosa tri-layer matrix compared with standard care in managing diabetic foot ulcers in the US | Patients with diabetic foot ulcers | Adjunctive porcine small intestine submucosa tri-layer matrix | 1 year | Markov | Healed, uninfected ulcer, infected, amputation, post amputation, gangrene, deceased | US payers | Costs, ulcer-free months |
| 11 | Epstein 2018 ^34^ | Cost-effectiveness of treatments for superficial venous reflux in patients with chronic venous ulceration | Patients with chronic venous ulceration | Superficial venous reflux | Lifetime | Markov | Unhealed ulcer, 1st year after healing, 2nd year after healing, 3rd year after healing, ulcer recurrence, dead | UK NHS and PSS | Cost per QALYs gained |
| 12 | Gueltzow 2018 ^35^ | Budget impact of antimicrobial wound dressings in the treatment of venous leg ulcers in the German outpatient care sector: a budget impact analysis | Patients with VLUs | Antimicrobial wound dressings | 1 year | Markov | Wound infection, no wound infection, healed, death | The German statutory health insurance | Budget impact (Cost) |
| 13 | Guest 2018 ^36^ | Cost-effectiveness of using a collagen-containing dressing plus compression therapy in non-healing venous leg ulcers | Patients with VLUs | Collagen-containing dressings | 6 months | Decision tree | Healed at one month, healed at two months, healed at three months, healed at four months, healed at five months, healed at six months, unhealed | UK NHS and PSS | Costs, QALYs |
| 14 | Walzer 2018 ^37^ | A cost-effectiveness analysis of a hydration response technology dressing in the treatment of venous leg ulcers in the UK | Patients with VLUs | Hydration response technology dressing | 1 year | Markov | Progressing ulcer, static/deteriorate ulcer, severe ulcer, healed | UK NHS and PSS | Costs, QALYs |
| 15 | Cheng 2018 ^38^ | Cost-effectiveness analysis of guideline-based optimal care for venous leg ulcers in Australia | Patients with VLUs | Guideline-based optimal care, usual care | 5 years | Markov | No VLU, unhealed VLU, complicated VLU with hospitalisation, healed, death | Societal perspective in Australian setting | Costs, QALYs |
| 16 | Ontario HTA Series ^39^ | Compression Stockings for the Prevention of Venous Leg Ulcer Recurrence: A Health Technology Assessment | People with VLUs | Compression stockings for prevention of VLU recurrence | 5 years | Markov | Healed ulcer, recurred ulcer, infected ulcer, dead | Payer's perspective | Costs, QALYs |
| 17 | Djalalov 2020 ^40^ | Economic evaluation of compression stockings for the prevention of venous leg ulcer recurrence in Ontario | People with healed VLUs | Compression stockings | 5 years | Markov | Healed ulcer, recurred ulcer, infected ulcer, dead | Ontario healthcare system | Costs, QALYs |
| 18 | Kirsner 2020 ^41^ | A Cost-Effectiveness Analysis Comparing Single-use and Traditional Negative Pressure Wound Therapy to Treat Chronic Venous and Diabetic Foot Ulcers | Patients with Chronic Venous and Diabetic Foot Ulcers | Negative Pressure Wound Therapy | 12 and 26 weeks | Markov | Open wound, closed wound | US payers | Costs, reduction of wounds per week |
| 19 | Rognoni 2020 ^42^ | Venous stenting for patients with outflow obstruction and leg ulcers: cost-effectiveness and budget impact analyses | Patients with outflow obstruction and leg ulcers | Venous stenting | 3 years | Markov | Active ulcer, healed, recurred ulcer | The Italian Healthcare Service | Costs, QALYs |
| 20 | Guest 2021 ^43^ | Potential cost-effectiveness of using adjunctive dehydrated human amnion/chorion membrane allograft in the management of non-healing diabetic foot ulcers in the United Kingdom | Patients with non-healing diabetic foot ulcers | Adjunctive dehydrated human amnion/chorion membrane allograft | 1 year | Markov | DFU, unchanged, improved, worsened, infection, amputation, post amputation, healed, recurred | UK NHS and PSS | Costs, QALYs |
| 21 | ONTARIO HTA SERIES ^44^ | Skin Substitutes for Adults With Diabetic Foot Ulcers and Venous Leg Ulcers: A Health Technology Assessment | Adults with Diabetic Foot Ulcers and VLUs | Skin Substitutes | 26 weeks | Markov | Unhealed, minor LEA (lower-extremity amputation), major LEA, post minor LEA, post major LEA, healed, death | Public payer's perspective | Costs, QALYs |
| 22 | Guest 2021 ^45^ | Cost-effectiveness of using intermittent pneumatic compression to manage hard-to-heal venous leg ulcers in the UK | Patients with VLUs | Intermittent pneumatic compression | 24 weeks | Markov | Uninfected-no improvement, infection, improved, healed | UK NHS and PSS | Costs, QALYs |
| 23 | Velickovic 2022 ^46^ | Cost-effectiveness analysis of superabsorbent wound dressings in patients with moderate-to-fullly exuding leg ulcers in Germany | Patients with moderate-to-fullly exuding leg ulcers | Superabsorbent wound dressing | 6 months | Markov | Healed, progressing ulcer, static ulcer, deteriorating ulcer, severe ulcer, death | German payers | Costs, healing rates, QALYs |
| 24 | Velickovic 2022 ^47^ | Superabsorbent wound dressings versus foams dressings for the management of moderate-to-fullly exuding venous leg ulcers in French settings: An early stage model-based economic evaluation | Patients with moderate-to-fullly exuding VLUs | Superabsorbent wound dressings, foams dressings | 24 weeks | Markov | Healed, progressing ulcer, static ulcer, deteriorating ulcer, severe ulcer, death | French national payer | Costs, QALYs |
| 25 | Guest 2022 ^48^ | Cost-effectiveness of Debrichem in managing hard-to-heal venous leg ulcers in the UK | Patients with hard-to-heal VLUs | The topical debriding agent | 1 year | Markov | Static ulcer, improved ulcer, infected ulcer, healed | UK NHS and PSS | Costs, QALYs |
| 26 | Zheng 2022 ^49^ | Cost-effectiveness of Compression Therapy With Early Endovenous Ablation in Venous Ulceration for a Medicare Population | Patients with VLUs | Early endovenous ablation of superficial venous reflux, | 3 years | Markov | Unhealed VLU, after VLU (healed), death | Payer's perspective | Costs, QALYs |
| 27 | Velickovic 2023 ^50^ | Superabsorbent Wound Dressing for Management of Patients With Moderate-to-Fullly Exuding Chronic Leg Ulcers: An Early Stage Model-Based Benefit-Harm Assessment | Patients With Moderate-to-Fullly Exuding Chronic Leg Ulcers | Superabsorbent Wound Dressing | 6 months | Markov | Healed, progressing, static, deteriorating, severe, death | NA | QALYs |
| 28 | Guest 2023 ^51^ | Relative cost-effectiveness of three compression bandages in treating newly diagnosed venous leg ulcers in the UK | Patients with VLUs | Two-layer cohesive compression bandage, two-layer compression system, cohesive inelastic bandage system | 1 year | Markov | Static ulcer, improved ulcer, infected ulcer, healed ulcer | UK NHS and PSS | Costs, QALYs |
| 29 | Cooper 2023 ^52^ | Cost-Effectiveness of PHMB & betaine wound bed preparation compared with standard care in venous leg ulcers: A cost-utility analysis in the United Kingdom | People with VLUs | Biofilm disrupting and cleansing solution and gel | 1 year | Markov | Open wound, closed wound, infected wound, death | UK NHS and PSS | Costs, QALYs |
| 30 | Guest 2023 ^53^ | Cost-effectiveness of two reduced pressure compression systems in treating newly diagnosed venous leg ulcers | Patients with VLUs | Reduced pressure compression systems | 1 year | Markov | Static ulcer, improved ulcer, infected ulcer, healed | UK NHS and PSS | Costs, QALYs |
| 31 | Guest 2024 ^54^ | Cost-effectiveness of ChloraSolv in treating hard-to-heal venous leg ulcers | Patients with hard-to-heal VLUs | Topical, amino acid-buffered hypochlorite debriding gel | 24 weeks | Markov | Static ulcer, improved ulcer, infected ulcer, healed | UK NHS and PSS | Costs, QALYs |
| 32 | CacuaSanchez 2024 ^55^ | Cost-effectiveness analysis of intralesional and perilesional recombinant human epidermal growth factor vs hydrocolloid therapy in venous ulcer treatment in the Colombian context | People with chronic venous insufficiency without infection | Intralesional and perilesional recombinant human epidermal growth factor (rhEGF), hydrocolloid therapy | 5 years | Markov | Ulcers, healed ulcers, infected ulcers, death | Health care system in Colombia | Costs, QALYs |

Abbreviations: NHS, National Health Services, PSS, Personal Social Services; QALYs, quality adjusted life years; VLU, venous leg ulcer;

Key identified model structures are presented in Figure 4.

Figure 4 Alternative decision model structures


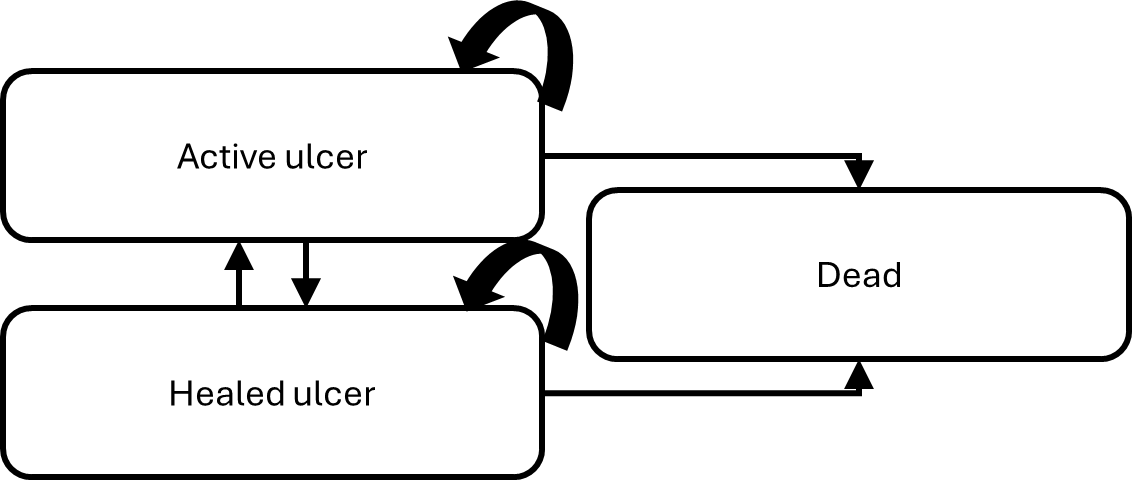


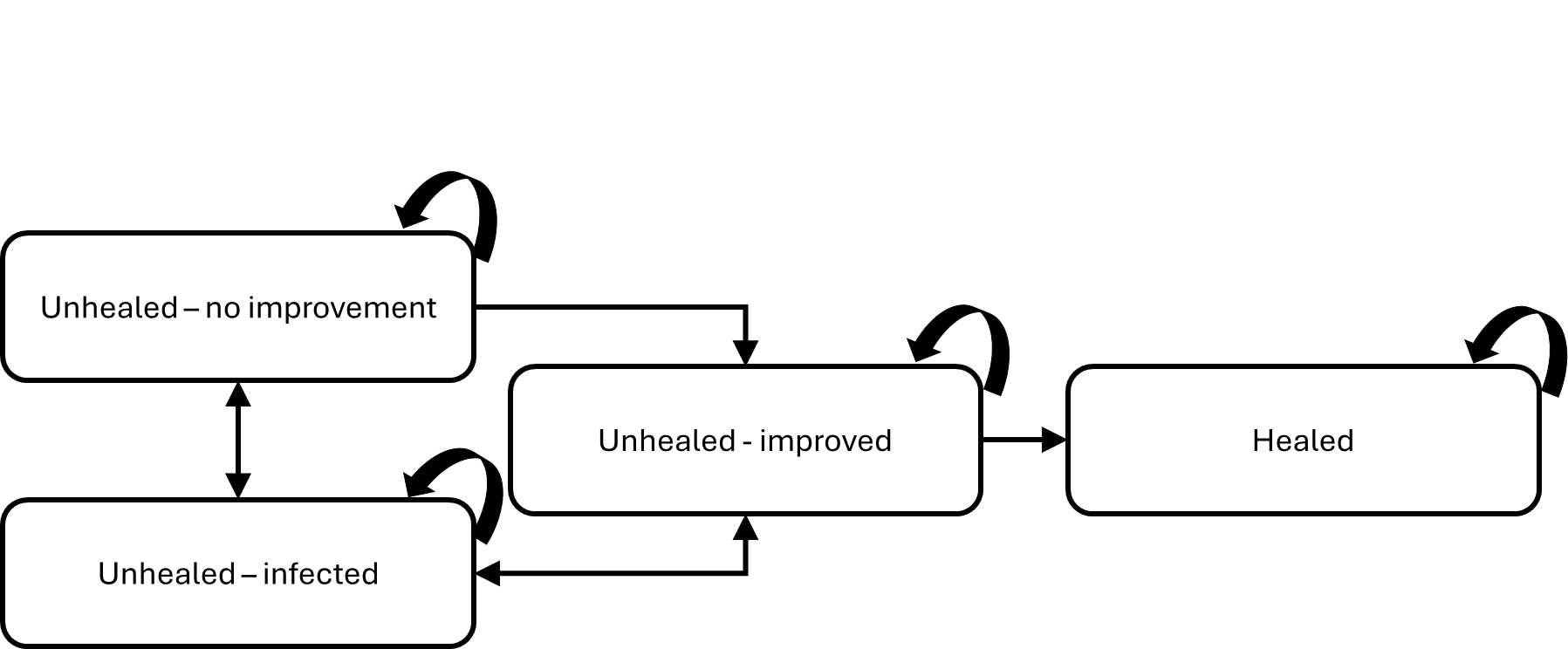

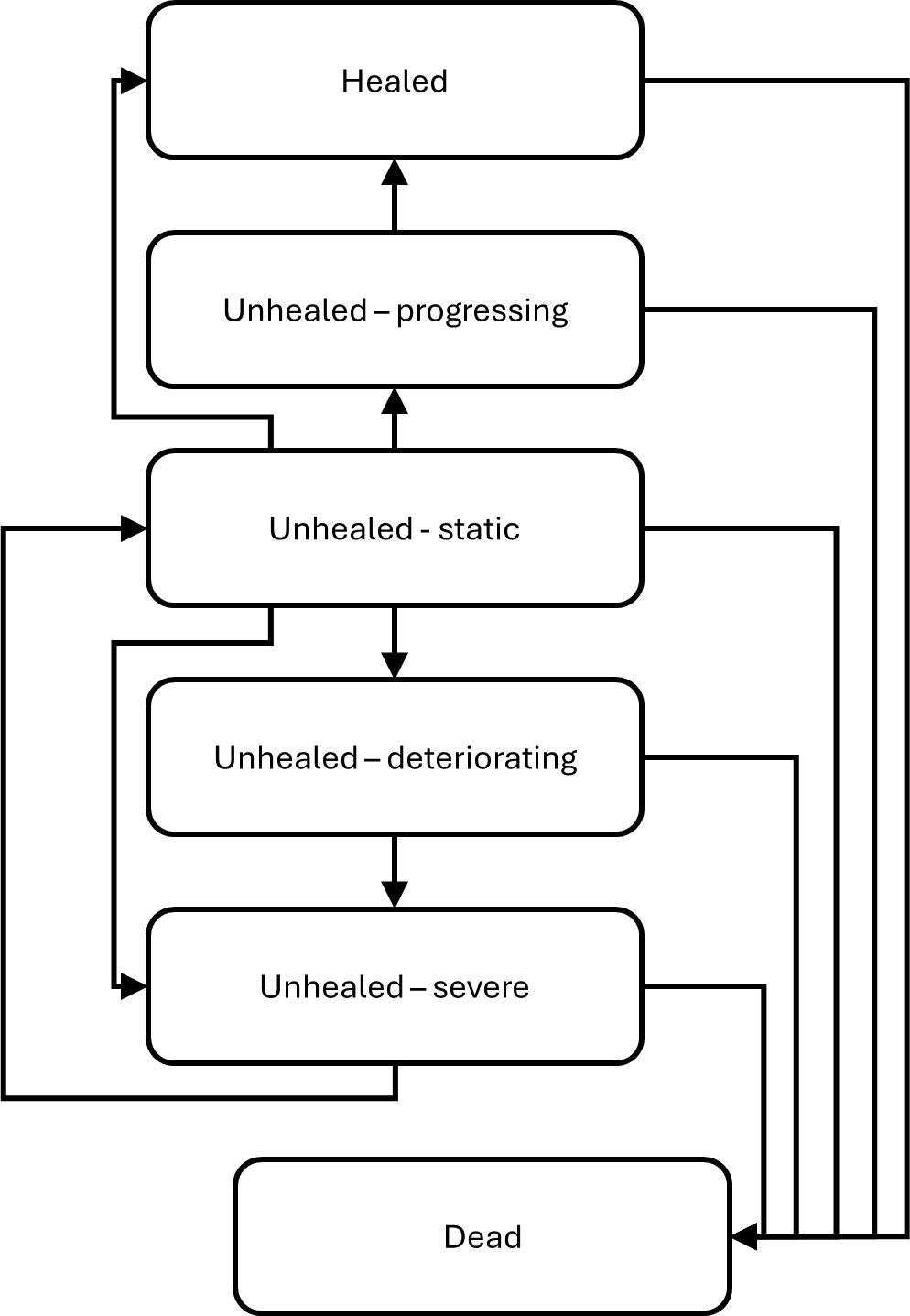

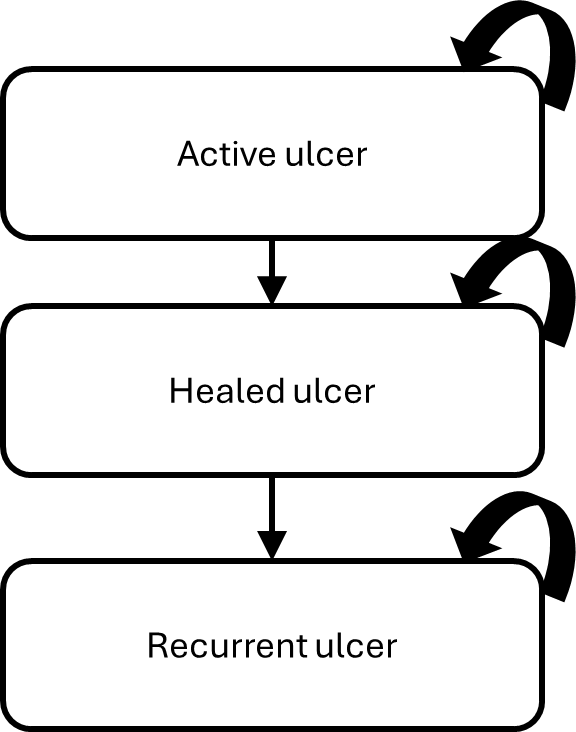


**Key findings from decision models available in literature:**

Infected ulcer was a common health state, though it was also modelled as an event. For example, in the Ontario HTA model ^39^ and the Carter 2014 model ^29^, the risk of infection was set at 10% within the first 3 months for unhealed ulcers, decreasing to 5% from month 4 onwards. Infection was associated with the cost of antibiotics (which was considered negligible and excluded from our analysis), a 10% risk of hospitalisation, with 20% of cases experiencing major complications and 80% having no complications. In all models, infection did not result in a reduction in utility. Ulcer infection is, however, not considered a disease modifier. In VenUS IV, difference in the number of infections, largely infection at wounds, was relatively small across two arms – 5.7% in 2LH and 6.9% in 4LB. VenUS 6 also found no statistical difference in the ulcer infection risk by 6- and 12-months post-randomisation across compressions. We therefore did not consider infection as a potential health state nor a major event in our economic model.

Ulcer progression was frequently modelled (e.g., static-improved-infected or static- progressing-deteriorating-severe). The primary aim for these models was to capture a proportion of ulcers that would never be healed. The literature suggests that a proportion of venous leg ulcers is hard or never healed. Albeit our model does not specifically define hard-to-heal ulcers, we account for this by using survival distributions that can effectively capture patients with these characteristics (i.e., via the use of a log-normal distribution).

# Appendix 7. Additional Detail on the Targeted Literature Reviews for Economic Model Parameterisation

The VenUS 6 provides comprehensive data on resource use, health-related quality of life, mortality, and recurrence, complementing the information from the VenUS I, VenUS IV, and EVRA trials.

Such data is regarded as high-level evidence, and specific for UK population. However, to ensure that the decision-analytic model incorporates all available evidence, we conducted targeted literature reviews to identify additional information for parameterisation. Such additional information, where appropriate, was used for scenario analyses to assess the robustness of cost-effectiveness outcomes.

Three targeted literature reviews were conducted in April 2023 using search terms from VenUS IV to identify recent (i.e., from 2011) relevant sources of mortality, resource use, and utility data. The search terms used in VenUS IV can be found elsewhere. ^12^

### Mortality Data

A total of 149 records related to mortality were identified and screened based on their abstracts. Two manuscripts were selected for full-text review: Salenius et al. 2021^56^ and Kreft et al. 2020^57^.

- Salenius 2021 ^56^: This single-centre retrospective cohort study in Finland found that individuals with venous leg ulcers had a fuller risk of death compared to the matched general population, with a hazard ratio (HR) of 1.49 (95% CI 1.41-1.59).
- Kreft 2020 ^57^: Utilizing health claims data from Germany’s largest public health insurer, Allgemeine Ortskrankenkasse (AOK), this study reported that individuals who have ever had venous leg ulceration had an increased mortality risk (HR 2.51, 95% CI 2.53-2.49) compared to those who never had.

Neither study provided information on the relative mortality risk between individuals with healed versus unhealed venous leg ulcers. Baseline characteristics in these two studies are not fully reported since they studied multiple types of ulceration.

### Resource Use Data

From 25 records found related to resource use, two studies were retrieved for full-text review: Pham et al. ^58^ and Carter et al. ^29^. However, since neither study was based in the UK, no data were extracted from them.

### Utility Data

A total of 89 citations related to utilities for individuals with venous leg ulcers were identified. After screening, seven full-text papers were obtained for detailed review (Guest et al. 2023, ^51^ Zheng et al. 2022, ^49^ Velickovic et al. 2022, ^47^ Cheng et al. 2019, ^59^ Barnsbee et al. 2019, ^60^ Balieva et al. 2017, ^61^ Yang et al. 2015 ^62^). None of these studies provided a more appropriate source for health state utility than the VenUS 6, which was based in the UK and uses the EQ-5D-5L instrument with utility scores estimated using the UK tariff. Instead, these studies serve to validate the utility parameters obtained from the VenUS 6 study externally (see Table 13).

Table 13 Utility values in identified studies from targeted literature review for utility

| **Studies** | **Setting of HRQoL study** | **Methods** | **Utility values** |
| --- | --- | --- | --- |
| **Guest 2023 ^51^** | United Kingdom | Standard gambles, general public | Static VLU: 0.64 (95% CI 0.61to 0.68)  Improving VLU: 0.73 (95% CI 0.70 to 0.76)  Healed VLU: 1.00 |
| **Zheng 2022 ^49^** | United States | EQ5D-5L, US tariff | Unhealed VLUs: 0.69  Healed VLUs: 0.75 (from VenUS I) |
| **Velickovic 2022 ^50^** | United Kingdom | Standard gambles, general public | Static or deteriorating VLU: 0.64 (95% CI 0.61 to 0.68)  Improving VLU: 0.73 (95% CI 0.70 to 0.76)  Healed VLU: 1.00  Severe VLU: 0.61 |
| **Cheng 2019 ^59^** | Australia | EQ5D-5L, UK tariff | Baseline:  All: 0.67 (SE: 0.24)  Responsiveness after 6 months:  Healed VLU: +0.14 (SE: 0.13)  Unhealed VLU: + 0.15 (SE: 0.18) |

Abbreviations: CI, confidence interval; HRQoL, health-related quality of life; SE, standard error; VLU, venous leg ulcer

### Conclusions on the targeted literature reviews for model parameterisation

The literature reviews on mortality could not identify relevant information to inform the difference in mortality rate for people with and without venous leg ulcers. We thus assumed that the mortality rates between healed and unhealed health states are similar.

Identified studies regarding utilities and resource use were deemed not applicable to our research. Our dataset, which includes IPD from VenUS I, VenUS IV, VenUS 6, and EVRA, appears to be most appropriate for the UK population and is thus primarily used to inform the majority of parameters in our model.

# Appendix 8. Supportive analyses for the assessment of inconsistency, heterogeneity, and certainty

In addition to the qualitative assessment of heterogeneity, consistency and transitivity assumption presented in the manuscript and Appendices 4 and 8, a reviewer requested a quantitative summary of the assessments, including:

- Funnel plots (where a sufficient number of studies were available)
- Heterogeneity, assessed using Tau squared, Cochrane’s Q test, *I*^2^ statistic
- Consistency, assessed using Cochran’s Q test
- A complete set of leave-one-out sensitivity analysis
- Assessment of evidence certainty using CINeMA^65^ ^66^

To be able to produce these outputs, the NMA was restructured (only for the purpose of this assessment) as follows:

- All data were analysed in aggregated format. The resulting dataset is presented in Table 2 of the manuscript.
- The outcome was defined as the number of healed ulcers during follow-up out of the number randomised, analysed using odds ratios on the log scale.
- A frequentist framework was adopted using the *netmeta* package in R.
- CINeMA^65^ ^66^ was perform using the freely available online platform (<https://cinema.med.auth.gr/>), with the clinically important effect size defined as 1.33, consistent with the non-inferiority margin used in VenUS 6.

Results from this NMA model are as follows:

Table 14 Supportive analyses: results of tests for heterogeneity and inconsistency using random-effects model

| **Tests** | **Output** |
| --- | --- |
| Tau^2^ (heterogeneity) | 0.1133 |
| I^2^ (heterogeneity) | 44.1% [0.0%; 70.8%] |
| Cochran’s Q test within designs (heterogeneity) | Q=18.14 (p= 0.0526) |
| Cochran’s Q test between designs (consistency) | Q=3.33 (p=0.1889) |

Table 15 Supportive analyses: Assessment of evidence certainty using CINeMA

| Comparison | # study | Within-study bias | Reporting bias | Indirectness | Imprecision | Heterogeneity | Incoherence | Confidence rating |
| --- | --- | --- | --- | --- | --- | --- | --- | --- |
| 2LB vs 4LB/2LH | 4 | No concerns | Low risk | No concerns | Major concerns | No concerns | No concerns | Moderate |
| 2LB vs CW | 2 | Some concerns | Low risk | No concerns | Major concerns | No concerns | No concerns | Moderate |
| 4LB/2LH vs CW | 1 | Some concerns | Low risk | No concerns | Major concerns | No concerns | No concerns | Moderate |
| 4LB/2LH vs SSB | 9 | Some concerns | Low risk | No concerns | Major concerns | No concerns | No concerns | Moderate |
| 2LB vs SSB | 0 | No concerns | Low risk | No concerns | Major concerns | No concerns | No concerns | Moderate |
| CW vs SSB | 0 | Some concerns | Low risk | No concerns | Major concerns | No concerns | No concerns | Moderate |

Figure 6 Supportive analyses: Comparison-adjusted funnel plot for comparison: 4LB/2LH vs SSB


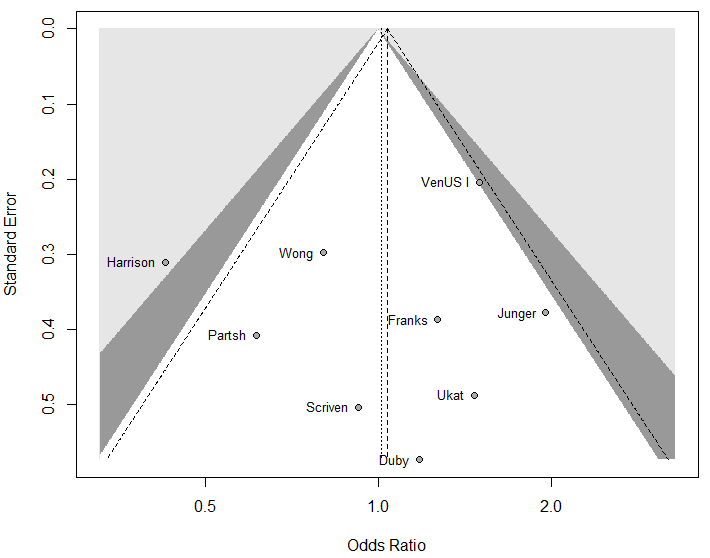


Figure 7 Supportive analyses: Comparison-adjusted funnel plot for comparison: 2LH vs 4LB/2LH


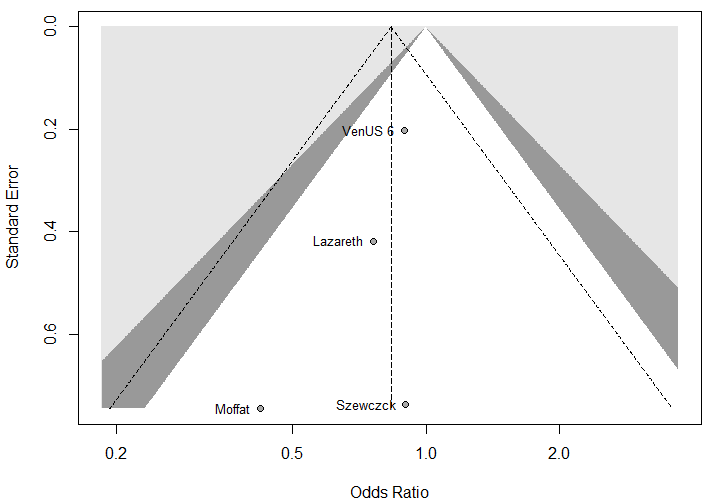


Table 16 Supportive analyses: Leave-one-out sensitivity results (OR vs 4LB/2LH)

| **Removed study** | **Treatment** | **OR vs 4LB/2LH** | **95% CI Lower bound** | **95% CI Upper bound** |
| --- | --- | --- | --- | --- |
| VenUS 6 | 2LB | 1.42 | 0.75 | 2.70 |
|  | CW | 2.30 | 0.63 | 8.33 |
|  | SSB | 0.96 | 0.76 | 1.20 |
| VenUS I | 2LB | 0.93 | 0.66 | 1.30 |
|  | CW | 0.82 | 0.56 | 1.20 |
|  | SSB | 1.12 | 0.86 | 1.47 |
| Duby 1993 | 2LB | 0.93 | 0.66 | 1.30 |
|  | CW | 0.82 | 0.56 | 1.20 |
|  | SSB | 0.96 | 0.76 | 1.21 |
| Scriven 1998 | 2LB | 0.93 | 0.66 | 1.30 |
|  | CW | 0.82 | 0.56 | 1.20 |
|  | SSB | 0.95 | 0.75 | 1.19 |
| Partsh 2001 | 2LB | 0.93 | 0.66 | 1.30 |
|  | CW | 0.82 | 0.56 | 1.20 |
|  | SSB | 0.91 | 0.72 | 1.15 |
| Ukat 2003 | 2LB | 0.93 | 0.66 | 1.30 |
|  | CW | 0.82 | 0.56 | 1.20 |
|  | SSB | 0.97 | 0.77 | 1.23 |
| Franks 2004 | 2LB | 0.93 | 0.66 | 1.30 |
|  | CW | 0.82 | 0.56 | 1.20 |
|  | SSB | 0.97 | 0.77 | 1.23 |
| Junger 2004b | 2LB | 0.93 | 0.66 | 1.30 |
|  | CW | 0.82 | 0.56 | 1.20 |
|  | SSB | 1.02 | 0.80 | 1.29 |
| Moffat 1996 | 2LB | 0.88 | 0.62 | 1.25 |
|  | CW | 0.80 | 0.55 | 1.17 |
|  | SSB | 0.96 | 0.76 | 1.20 |
| Szewczck 2010 | 2LB | 0.92 | 0.65 | 1.30 |
|  | CW | 0.82 | 0.56 | 1.20 |
|  | SSB | 0.96 | 0.76 | 1.20 |
| Wong 2012 | 2LB | 0.93 | 0.66 | 1.30 |
|  | CW | 0.82 | 0.56 | 1.20 |
|  | SSB | 0.91 | 0.72 | 1.16 |
| Harrison 2011 | 2LB | 0.93 | 0.66 | 1.30 |
|  | CW | 0.82 | 0.56 | 1.20 |
|  | SSB | 0.83 | 0.65 | 1.06 |
| Mosti 2020 | 2LB | 0.95 | 0.68 | 1.35 |
|  | CW | 0.79 | 0.54 | 1.16 |
|  | SSB | 0.96 | 0.76 | 1.20 |
| Lazareth 2012 | 2LB | 0.86 | 0.59 | 1.25 |
|  | CW | 0.79 | 0.54 | 1.16 |
|  | SSB | 0.96 | 0.76 | 1.20 |

# Appendix 9. Additional Results of Scenario Analysis for the NMA

In the main analysis, a log-normal distribution was used for the modelling of time to ulcer healing with a fixed-effect model. HR estimates from this model are presented in the main text. Table 17 presents median time to ulcer healing estimated from this model, using a mean ulcer size of 24.8 cm^2^, and mean ulcer duration of 10.0 months (baseline characteristics of participants in VenUS 6).

Table 17 Median time to ulcer healing, fixed-effects model with a log-normal distribution

| Treatments | Median time to ulcer healing, months (95% CrI) |
| --- | --- |
| 4LB/2LH | 2.53 (2.25 to 2.82) |
| SSB | 2.56 (2.24 to 2.87) |
| 2LB | 2.50 (2.22 to 2.77) |
| CW | 2.62 (2.24 to 2.90) |

We here present the results for other scenarios assuming a random-effect approach and a log-logistic or a Weibull distribution for the synthesis of time to ulcer healing data (see Table 18). We also present the NMA results for the main analysis of all treatments included in the evidence Network 1. For discussion on the importance of VenUS 6, we provide NMA results for the hypothetical scenario in which VenUS 6 was assumed not available.

No adjustment for multi-arm studies is needed in our base case analysis, which used a fixed effects with arm-level data. ^63^ Where we have used random-effects model, we could not implement such adjustment because the methods to do this for multi-arm studies in NMAs combining individual patient and aggregated data are not yet available. We note that as the credible intervals for all treatment effects encompassed zero, the implementation of such adjustment (that would likely widen credible intervals) is unlikely to alter our conclusions.

Table 18 Network meta-analysis scenario results

|  | **Random-effects** | | | | **Alternative survival distributions** | | | | **Excluding VenUS 6** |  |
| --- | --- | --- | --- | --- | --- | --- | --- | --- | --- | --- |
|  | **Network 1**  **Random-effects** | | **Network 2**  **Random-effects** | | **Log-logistic** | | **Weibull** | |  |  |
| **HR vs 4LB/2LH** | |  | |  | |  | |  | | |
| SSB | 0.997 (0.782 to 1.247) | | 0.984 (0.651 to 1.415) | | 0.934 (0.808 to 1.071) | | 1.004 (0.885 to 1.141) | | 0.974 (0.853 to 1.105) |  |
| 2LB | 1.081 (0.751 to 1.521) | | 1.024 (0.576 to 1.682) | | 1.013 (0.877 to 1.168) | | 1.055 (0.868 to 1.277) | | 1.261 (0.856 to 1.785) |  |
| CW | 1.035 (0.623 to 1.675) | | 1.063 (0.518 to 2.072) | | 0.979 (0.843 to 1.132) | | 0.910 (0.723 to 1.127) | | 1.801 (0.758 to 3.627) |  |
| **SUCRA** |  | |  | |  | |  | |  |  |
| 4LB/2LH | 46% | | 55% | | 51% | | 49% | | 36% |  |
| SSB | 44% | | 47% | | 36% | | 49% | | 32% |  |
| 2LB | 53% | | 52% | | 53% | | 57% | | 59% |  |
| CW | 46% | | 53% | | 45% | | 33% | | 75% |  |

Abbreviations: HR, hazard ratio; SUCRA, surface uncer the cumulative ranking curve

Table 19 provides the consistency matrix for NMA model results, where the upper diagonal of the matrix reports the direct estimates (see below for further detail), and the lower diagonal reports the pooled relative effect estimates derived from the NMA model. The direct estimates were obtained:

- Directly extracted from studies where available;
- When the HR was not reported in the study, and when there was only one study informing the pairwise, the HR was calculated as the ratio between the two odds.^64^
- When there were two or more studies informing a contrast, a meta-analysis assuming fixed-effects was conducted. Pairwise comparisons with only AD were pooled using a fixed-effect model for the synthesis of hazard ratios. Pairwise comparisons with IPD and AD were pooled using a fixed-effect joint model of IPD and AD (assuming a Log normal distribution).

Table 19 Consistency matrix for network meta-analysis results of all included treatments – hazard ratios of time to healing

|  | Direct estimates | | | | | | | | | | |
| --- | --- | --- | --- | --- | --- | --- | --- | --- | --- | --- | --- |
| Indirect estimate | 4LB/2LH | 0.99 ^a^  [0.87 , 1.24] | 1.24 ^b^  [0.38 , 4.06] | 0.62 ^c^  [0.33 , 1.15] | 0.9 ^c^  [0.19 , 4.16] | 1.56 ^c^  [0.38 , 6.3] | 1 ^c^  [0.2 , 4.95] | 1.05^d^  [0.90 , 1.41] | 2.92 ^c^  [1.25 , 6.82] | NA | 0.78 ^b^  [0.61 , 0.99] |
|  | 0.97  [0.85 , 1.11] | SSB | NA | NA | NA | NA | NA | NA | NA | NA | NA |
|  | 1.26  [0.49 , 2.66] | 1.3  [0.51 , 2.76] | Ba | NA | NA | NA | NA | NA | NA | NA | NA |
|  | 0.8  [0.39 , 1.47] | 0.83  [0.39 , 1.54] | 0.77  [0.22 , 1.94] | Paste | NA | NA | NA | NA | NA | NA | NA |
|  | 1.04  [0.39 , 2.22] | 1.08  [0.4 , 2.32] | 1  [0.24 , 2.76] | 1.46  [0.42 , 3.77] | BHeH | NA | NA | NA | NA | NA | NA |
|  | 1.61  [0.4 , 4.5] | 1.67  [0.41 , 4.7] | 1.54  [0.27 , 5.03] | 2.26  [0.44 , 6.98] | 1.88  [0.33 , 6.34] | BzeaH | NA | NA | NA | NA | NA |
|  | 1.17  [0.32 , 3.06] | 1.21  [0.33 , 3.14] | 1.11  [0.22 , 3.44] | 1.65  [0.35 , 4.92] | 1.37  [0.26 , 4.41] | 1.05  [0.15 , 3.77] | HV | NA | NA | NA | NA |
|  | 1.04  [0.86 , 1.26] | 1.08  [0.84 , 1.35] | 1  [0.38 , 2.14] | 1.47  [0.69 , 2.78] | 1.22  [0.45 , 2.69] | 0.95  [0.23 , 2.62] | 1.24  [0.33 , 3.29] | 2LB | NA | NA | 0.79 ^d^  [0.61 , 1.01] |
|  | 2.11  [1.14 , 3.63] | 2.18  [1.15 , 3.8] | 2.01  [0.65 , 4.94] | 2.96  [1.1 , 6.55] | 2.47  [0.75 , 6.25] | 1.91  [0.39 , 5.75] | 2.5  [0.58 , 7.19] | 2.04  [1.07 , 3.62] | r2LB | NA | NA |
|  | 0.89  [0.34 , 1.91] | 0.92  [0.35 , 2.01] | 0.85  [0.21 , 2.39] | 1.25  [0.36 , 3.26] | 1.04  [0.25 , 2.93] | 0.81  [0.13 , 2.72] | 1.05  [0.19 , 3.31] | 0.86  [0.33 , 1.85] | 0.46  [0.14 , 1.13] | SSB/2LB | 1.23 ^c^  [0.44 , 3.43] |
|  | 0.93  [0.74 , 1.14] | 0.96  [0.74 , 1.22] | 0.88  [0.33 , 1.89] | 1.3  [0.6 , 2.47] | 1.08  [0.4 , 2.42] | 0.84  [0.2 , 2.32] | 1.1  [0.3 , 2.94] | 0.89  [0.72 , 1.09] | 0.48  [0.24 , 0.83] | 1.25  [0.5 , 2.61] | CW |

^a^, estimates from a meta-analysis with fixed-effect model, comprising of IPD and AD (Log normal distribution)

^b^, estimates from a single trial with IPD

^c^, estimates from single trial with aggregated data

^d^, estimates from a meta-analysis with fixed-effect model

NA: direct evidence not available

For direct estimates, HRs greater than 1 favour column-defining treatments; for indirect estimates, HRs greater than 1 favour row-defining treatments

# Appendix 10. Additional Results of Scenario Analyses for the Cost-Effectiveness Model

Table 20 List of scenario analyses to assess the robustness of the cost-effectiveness results

| **Items** | **Base case** | **Scenario** | **Result table** |
| --- | --- | --- | --- |
| Fixed effect vs random effect NMA model | Fixed effect | Network meta-analysis considering a random effect with predictive distributions | Table 21 |
| Alternative survival distributions for time to healing | Log-normal | Log-logistic | Table 22 |
| Alternative survival distributions for time to healing | Log-normal | Weibull | Table 23 |
| Alternative NMA evidence networks | Network 1 | Network 2 | Table 24 |
| Alternative NMA evidence networks | Network 1 | Network 3 | Table 25 |
| Model structure | Recurrence rate depends on time patients spent healed | Fixed recurrence rate within the first two years | Table 26 |
| Model structure | Recurrence rate depends on time patients spent healed | Recurrence rate is time dependent, but not dependent of time spent healed | Table 27 |
| Exceed mortality rate | Sourced from VenUS 6 (1.79) | The same as VenUS IV (2.36) | Table 28 |

Abbreviations: NMA, network meta-analysis

Table 21 Cost-effectiveness results with a random effect NMA with predictive distributions informing the effectiveness of treatments on time to ulcer healing

| **Treatment** | **4LB/2LH** | **SSB** | **2LB** | **CW** |
| --- | --- | --- | --- | --- |
| **Total costs £**  **(mean, 95% CI)** | 2596 (1030 to 5807) | 3136 (1068 to 8035) | 3089 (1030 to 8050) | 2923 (911 to 7591) |
| **Total QALYs**  **(mean, 95% CI)** | 6.49 (6.03 to 6.94) | 6.49 (6.03 to 6.93) | 6.49 (6.03 to 6.95) | 6.49 (6.01 to 6.93) |
| **Inc. costs £**  **(mean, 95% CI)** | Reference treatment | 540 (-1141 to 3515) | 493 (-1328 to 3786) | 327 (-1616 to 3479) |
| **Inc. QALYs**  **(mean, 95% CI)** | Reference treatment | -0.0048  (-0.065 to 0.0359) | 0.001  (-0.0671 to 0.04) | -0.0036  (-0.08 to 0.0442) |
| **Mean ICER**  **(£/QALY gained)** | Reference treatment | Dominated ^b^ | 493010 | Dominated ^b^ |
| **Net monetary benefit (NMB) ^a^** | 127226  (117491 to 136174) | 126589  (115683 to 136017) | 126713  (115948 to 136207) | 126826  (116257 to 136614) |
| **Inc. NMB ^c^** | Reference treatment | -637 (-4635 to 1941) | -513 (-4965 to 2164) | -400 (-5150 to 2507) |

^a^ at a cost-effectiveness threshold of £20,000/QALY gained. A positive incremental net monetary benefit implies that the treatment is cost-effective; ^b^ Dominated: the treatment is more expensive and offers less health benefits than the reference treatment; ^c^ Incremental NMB indicates the differences in NMB of comparators against the reference treatment; positive values indicate that the treatment is cost-effective and negative values the opposite.

Abbreviations: ICER, incremental cost-effectiveness ratio; QALYs, quality-adjusted life years; NMB, net monetary benefits; CI, confidence interval

Table 22 Cost-effectiveness results with the NMA considering a Log logistic survival distribution

| **Treatment** | **2LH/4LB** (costed as EBC) | **SSB** | **2LB** | **CW** |
| --- | --- | --- | --- | --- |
| **Total costs £**  **(mean, 95% CI)** | 3750 (2771 to 6374) | 4360 (3271 to 7018) | 4382 (3328 to 6976) | 4026 (2962 to 6705) |
| **Total QALYs**  **(mean, 95% CI)** | 6.5 (6.14 to 6.85) | 6.49 (6.14 to 6.84) | 6.5 (6.14 to 6.85) | 6.49 (6.14 to 6.85) |
| **Inc. costs £**  **(mean, 95% CI)** | Reference treatment | 610 (287 to 999) | 632 (334 to 960) | 275 (5 to 568) |
| **Inc. QALYs**  **(mean, 95% CI)** | Reference treatment | -0.0032 (-0.0114 to 0.0032) | 0.0006  (-0.0057 to 0.01) | -0.0009  (-0.0075 to 0.0061) |
| **Mean ICER**  **(£/QALY gained)** | Reference treatment | Dominated ^b^ | 1128854 | Dominated ^b^ |
| **Net monetary benefit (NMB) ^a^** | 126155  (118812 to 133387) | 125481  (118198 to 132709) | 125534  (118211 to 132850) | 125861  (118436 to 133128) |
| **Inc. NMB ^c^** | Reference treatment | -674 (-1214 to -243) | -621 (-1041 to -211) | -293 (-689 to 100) |

^a^ at a cost-effectiveness threshold of £20,000/QALY gained. A positive incremental net monetary benefit implies that the treatment is cost-effective; ^b^ Dominated: the treatment is more expensive and offers less health benefits than the reference treatment; ^c^ Incremental NMB indicates the differences in NMB of comparators against the reference treatment; positive values indicate that the treatment is cost-effective and negative values the opposite.

Abbreviations: ICER, incremental cost-effectiveness ratio; QALYs, quality-adjusted life years; NMB, net monetary benefits; CI, confidence interval.

Table 23 Cost-effectiveness results with the NMA considering a Weibull survival distribution

| **Treatment** | **2LH/4LB** (costed as EBC) | **SSB** | **2LB** | **CW** |
| --- | --- | --- | --- | --- |
| **Total costs £**  **(mean, 95% CI)** | 2242 (809 to 5417) | 2525 (931 to 6188) | 2528 (989 to 5762) | 2572 (985 to 6049) |
| **Total QALYs**  **(mean, 95% CI)** | 6.53 (6.17 to 6.88) | 6.53 (6.17 to 6.88) | 6.53 (6.18 to 6.88) | 6.52 (6.16 to 6.87) |
| **Inc. costs £**  **(mean, 95% CI)** | Reference treatment | 284 (9 to 796) | 286 (-98 to 829) | 331 (-152 to 1005) |
| **Inc. QALYs**  **(mean, 95% CI)** | Reference treatment | -0.0001  (-0.0072 to 0.0063) | 0.0023  (-0.0061 to 0.01) | -0.0044  (-0.0188 to 0.0078) |
| **Mean ICER**  **(£/QALY gained)** | Reference treatment | Dominated ^b^ | 124948 | Dominated ^b^ |
| **Net monetary benefit (NMB) ^a^** | 128334  (120475 to 135966) | 128049  (119973 to 135659) | 128093  (120232 to 135831) | 127915  (119713 to 135549) |
| **Inc. NMB ^c^** | Reference treatment | -285 (-907 to 94) | -240 (-929 to 337) | -419 (-1362 to 305) |

^a^ at a cost-effectiveness threshold of £20,000/QALY gained. A positive incremental net monetary benefit implies that the treatment is cost-effective; ^b^ Dominated: the treatment is more expensive and offers less health benefits than the reference treatment; ^c^ Incremental NMB indicates the differences in NMB of comparators against the reference treatment; positive values indicate that the treatment is cost-effective and negative values the opposite.

Abbreviations: ICER, incremental cost-effectiveness ratio; QALYs, quality-adjusted life years; NMB, net monetary benefits; CI, confidence interval.

Table 24 Cost-effectiveness results with a NMA with low and moderate RoB studies (network 2)

| **Treatment** | **2LH/4LB** (costed as EBC) | **SSB** | **2LB** | **CW** |
| --- | --- | --- | --- | --- |
| **Total costs £**  **(mean, 95% CI)** | 2443 (1035 to 5086) | 2874 (1225 to 6132) | 2853 (1235 to 5933) | 2810 (1200 to 5739) |
| **Total QALYs**  **(mean, 95% CI)** | 6.53 (6.16 to 6.89) | 6.53 (6.15 to 6.89) | 6.53 (6.16 to 6.89) | 6.52 (6.15 to 6.89) |
| **Inc. costs £**  **(mean, 95% CI)** | Reference treatment | 431 (83 to 1162) | 411 (-70 to 1174) | 367 (-89 to 1126) |
| **Inc. QALYs**  **(mean, 95% CI)** | Reference treatment | -0.0028  (-0.0134 to 0.0046) | 0.0004  (-0.012 to 0.01) | -0.005  (-0.0213 to 0.007) |
| **Mean ICER**  **(£/QALY gained)** | Reference treatment | Dominated ^b^ | 1125645 | Dominated ^b^ |
| **Net monetary benefit (NMB) ^a^** | 128125  (120242 to 135690) | 127638  (119359 to 135258) | 127722  (119646 to 135339) | 127658  (119629 to 135325) |
| **Inc. NMB ^c^** | Reference treatment | -487 (-1398 to -16) | -403 (-1413 to 271) | -467 (-1541 to 248) |

^a^ at a cost-effectiveness threshold of £20,000/QALY gained. A positive incremental net monetary benefit implies that the treatment is cost-effective; ^b^ Dominated: the treatment is more expensive and offers less health benefits than the reference treatment; ^c^ Incremental NMB indicates the differences in NMB of comparators against the reference treatment; positive values indicate that the treatment is cost-effective and negative values the opposite.

Abbreviations: ICER, incremental cost-effectiveness ratio; QALYs, quality-adjusted life years; NMB, net monetary benefits; CI, confidence interval.

Table 25 Cost-effectiveness results with a NMA with low RoB studies (network 3)

| **Treatment** | **2LH/4LB** (costed as EBC) | **SSB** | **2LB** | **CW** |
| --- | --- | --- | --- | --- |
| **Total costs £**  **(mean, 95% CI)** | 2373 (1051 to 5070) | 2673 (1166 to 5608) | 2816 (1281 to 6018) | 2866 (1236 to 6092) |
| **Total QALYs**  **(mean, 95% CI)** | 6.52 (6.19 to 6.88) | 6.52 (6.19 to 6.88) | 6.52 (6.19 to 6.88) | 6.52 (6.19 to 6.88) |
| **Inc. costs £**  **(mean, 95% CI)** | Reference treatment | 301 (-71 to 872) | 443 (-65 to 1178) | 493 (-5 to 1298) |
| **Inc. QALYs**  **(mean, 95% CI)** | Reference treatment | -0.0001  (-0.0103 to 0.0097) | -0.0005  (-0.0136 to 0.01) | -0.0083  (-0.0263 to 0.0035) |
| **Mean ICER**  **(£/QALY gained)** | Reference treatment | Dominated ^b^ | Dominated ^b^ | Dominated ^b^ |
| **Net monetary benefit (NMB) ^a^** | 128121  (120794 to 135954) | 127820  (120190 to 135642) | 127667  (119716 to 135393) | 127463  (119600 to 135217) |
| **Inc. NMB ^c^** | Reference treatment | -302 (-1076 to 222) | -454 (-1431 to 290) | -658 (-1775 to 66) |

^a^ at a cost-effectiveness threshold of £20,000/QALY gained. A positive incremental net monetary benefit implies that the treatment is cost-effective; ^b^ Dominated: the treatment is more expensive and offers less health benefits than the reference treatment; ^c^ Incremental NMB indicates the differences in NMB of comparators against the reference treatment; positive values indicate that the treatment is cost-effective and negative values the opposite.

Abbreviations: ICER, incremental cost-effectiveness ratio; QALYs, quality-adjusted life years; NMB, net monetary benefits; CI, confidence interval.

Table 26 Cost-effectiveness results with a fixed recurrence rate from VenUS 6 within the first two years

| **Treatment** | **2LH/4LB** (costed as EBC) | **SSB** | **2LB** | **CW** |
| --- | --- | --- | --- | --- |
| **Total costs £**  **(mean, 95% CI)** | 3350 (1639 to 7006) | 3834 (1896 to 7793) | 3833 (1867 to 7683) | 3751 (1820 to 7842) |
| **Total QALYs**  **(mean, 95% CI)** | 6.53 (6.17 to 6.9) | 6.53 (6.16 to 6.9) | 6.53 (6.17 to 6.9) | 6.53 (6.17 to 6.89) |
| **Inc. costs £**  **(mean, 95% CI)** | Reference treatment | 484 (121 to 1094) | 483 (39 to 1195) | 401 (-96 to 1223) |
| **Inc. QALYs**  **(mean, 95% CI)** | Reference treatment | -0.0015  (-0.0104 to 0.0062) | 0.0022  (-0.0095 to 0.01) | -0.0045  (-0.02 to 0.0078) |
| **Mean ICER**  **(£/QALY gained)** | Reference treatment | Dominated ^b^ | 221561 | Dominated ^b^ |
| **Net monetary benefit (NMB) ^a^** | 127300 (119221 to 135044) | 126785  (118724 to 134682) | 126861  (118537 to 134706) | 126808  (118486 to 134750) |
| **Inc. NMB ^c^** | Reference treatment | -515 (-1253 to -24) | -439 (-1362 to 204) | -492 (-1661 to 227) |

^a^ at a cost-effectiveness threshold of £20,000/QALY gained. A positive incremental net monetary benefit implies that the treatment is cost-effective; ^b^ Dominated: the treatment is more expensive and offers less health benefits than the reference treatment; ^c^ Incremental NMB indicates the differences in NMB of comparators against the reference treatment; positive values indicate that the treatment is cost-effective and negative values the opposite.

Abbreviations: ICER, incremental cost-effectiveness ratio; QALYs, quality-adjusted life years; NMB, net monetary benefits; CI, confidence interval.

Table 27 Cost-effectiveness results with a time dependent recurrence rate, but not dependent of time patients spent healed

| **Treatment** | **2LH/4LB** (costed as EBC) | **SSB** | **2LB** | **CW** |
| --- | --- | --- | --- | --- |
| **Total costs £**  **(mean, 95% CI)** | 3117 (1320 to 6447) | 3577 (1507 to 7247) | 3549 (1567 to 7258) | 3511 (1551 to 7323) |
| **Total QALYs**  **(mean, 95% CI)** | 6.5 (6.07 to 6.97) | 6.5 (6.06 to 6.97) | 6.51 (6.06 to 6.97) | 6.5 (6.06 to 6.97) |
| **Inc. costs £**  **(mean, 95% CI)** | Reference treatment | 460 (84 to 1033) | 432 (-58 to 1199) | 395 (-83 to 1161) |
| **Inc. QALYs**  **(mean, 95% CI)** | Reference treatment | -0.0016  (-0.0113 to 0.0063) | 0.0023  (-0.009 to 0.02) | -0.0048  (-0.0195 to 0.0086) |
| **Mean ICER**  **(£/QALY gained)** | Reference treatment | Dominated ^b^ | 184232 | Dominated ^b^ |
| **Net monetary benefit (NMB) ^a^** | 126944 (117134 to 136608) | 126451  (116638 to 136085) | 126559  (116725 to 136241) | 126454  (116446 to 136361) |
| **Inc. NMB ^c^** | Reference treatment | -493 (-1276 to 37) | -385 (-1372 to 324) | -490 (-1564 to 269) |

^a^ at a cost-effectiveness threshold of £20,000/QALY gained. A positive incremental net monetary benefit implies that the treatment is cost-effective; ^b^ Dominated: the treatment is more expensive and offers less health benefits than the reference treatment; ^c^ Incremental NMB indicates the differences in NMB of comparators against the reference treatment; positive values indicate that the treatment is cost-effective and negative values the opposite.

Abbreviations: ICER, incremental cost-effectiveness ratio; QALYs, quality-adjusted life years; NMB, net monetary benefits; CI, confidence interval.

Table 28 Cost-effectiveness results considering the same excess mortality rate as VenUS IV (2.36)

| **Treatment** | **2LH/4LB** (costed as EBC) | **SSB** | **2LB** | **CW** |
| --- | --- | --- | --- | --- |
| **Total costs £ (mean, 95% CI)** | 2542 (1079 to 5600) | 2925 (1241 to 6263) | 2885 (1233 to 6032) | 2879 (1212 to 6148) |
| **Total QALYs (mean, 95% CI)** | 5.83 (5.42 to 6.26) | 5.83 (5.42 to 6.26) | 5.83 (5.42 to 6.26) | 5.82 (5.42 to 6.25) |
| **Inc. costs £ (mean, 95% CI)** | Reference treatment | 383 (47 to 955) | 344 (-88 to 1089) | 338 (-124 to 1035) |
| **Inc. QALYs (mean, 95% CI)** | Reference treatment | -0.0014 (-0.0108 to 0.0062) | 0.0021 (-0.0082 to 0.01) | -0.0043 (-0.0201 to 0.0076) |
| **Mean ICER (£/QALY gained)** | Reference treatment | Dominated ^b^ | 162662 | Dominated ^b^ |
| **Net monetary benefit (NMB) ^a^** | 113997 (104942 to 123048) | 113586 (104427 to 122625) | 113696 (104619 to 122729) | 113574 (104448 to 122554) |
| **Inc. NMB ^c^** | Reference treatment | -412 (-1140 to 61) | -302 (-1186 to 325) | -423 (-1550 to 257) |

^a^ at a cost-effectiveness threshold of £20,000/QALY gained. A positive incremental net monetary benefit implies that the treatment is cost-effective; ^b^ Dominated: the treatment is more expensive and offers less health benefits than the reference treatment; ^c^ Incremental NMB indicates the differences in NMB of comparators against the reference treatment; positive values indicate that the treatment is cost-effective and negative values the opposite.

Abbreviations: ICER, incremental cost-effectiveness ratio; QALYs, quality-adjusted life years; NMB, net monetary benefits; CI, confidence interval.

# Appendix 11. PRISMA and PRISMA-NMA checklists

| **Section and Topic** | **Item #** | **Checklist item** | **Location where item is reported** |
| --- | --- | --- | --- |
| **TITLE** | | |  |
| Title | 1 | Identify the report as a systematic review. | Title p1 |
| **ABSTRACT** | | |  |
| Abstract | 2 | See the PRISMA 2020 for Abstracts checklist. |  |
| **INTRODUCTION** | | |  |
| Rationale | 3 | Describe the rationale for the review in the context of existing knowledge. | Intro p5 |
| Objectives | 4 | Provide an explicit statement of the objective(s) or question(s) the review addresses. | Intro p5 |
| **METHODS** | | |  |
| Eligibility criteria | 5 | Specify the inclusion and exclusion criteria for the review and how studies were grouped for the syntheses. | Meth p6, App 3 p3-4 |
| Information sources | 6 | Specify all databases, registers, websites, organisations, reference lists and other sources searched or consulted to identify studies. Specify the date when each source was last searched or consulted. | App 3 p3-4 |
| Search strategy | 7 | Present the full search strategies for all databases, registers and websites, including any filters and limits used. | App 3 p3-4 |
| Selection process | 8 | Specify the methods used to decide whether a study met the inclusion criteria of the review, including how many reviewers screened each record and each report retrieved, whether they worked independently, and if applicable, details of automation tools used in the process. | App 3 p3-4 |
| Data collection process | 9 | Specify the methods used to collect data from reports, including how many reviewers collected data from each report, whether they worked independently, any processes for obtaining or confirming data from study investigators, and if applicable, details of automation tools used in the process. | App 3 p3-4 |
| Data items | 10a | List and define all outcomes for which data were sought. Specify whether all results that were compatible with each outcome domain in each study were sought (e.g. for all measures, time points, analyses), and if not, the methods used to decide which results to collect. | Methods p6 App 3 p3-4 |
|  | 10b | List and define all other variables for which data were sought (e.g. participant and intervention characteristics, funding sources). Describe any assumptions made about any missing or unclear information. | Methods p6 |
| Study risk of bias assessment | 11 | Specify the methods used to assess risk of bias in the included studies, including details of the tool(s) used, how many reviewers assessed each study and whether they worked independently, and if applicable, details of automation tools used in the process. | Methods p7, App 3 p3-4 |
| Effect measures | 12 | Specify for each outcome the effect measure(s) (e.g. risk ratio, mean difference) used in the synthesis or presentation of results. | Methods p7, App 4 p5 |
| Synthesis methods | 13a | Describe the processes used to decide which studies were eligible for each synthesis (e.g. tabulating the study intervention characteristics and comparing against the planned groups for each synthesis (item #5)). | NA |
|  | 13b | Describe any methods required to prepare the data for presentation or synthesis, such as handling of missing summary statistics, or data conversions. | NA |
|  | 13c | Describe any methods used to tabulate or visually display results of individual studies and syntheses. | Methods p7 |
|  | 13d | Describe any methods used to synthesize results and provide a rationale for the choice(s). If meta-analysis was performed, describe the model(s), method(s) to identify the presence and extent of statistical heterogeneity, and software package(s) used. | Methods p8, App 4 p5-8 |
|  | 13e | Describe any methods used to explore possible causes of heterogeneity among study results (e.g. subgroup analysis, meta-regression). | NA |
|  | 13f | Describe any sensitivity analyses conducted to assess robustness of the synthesized results. | Methods p7 |
| Reporting bias assessment | 14 | Describe any methods used to assess risk of bias due to missing results in a synthesis (arising from reporting biases). | NA |
| Certainty assessment | 15 | Describe any methods used to assess certainty (or confidence) in the body of evidence for an outcome. | NA |
| **RESULTS** | | |  |
| Study selection | 16a | Describe the results of the search and selection process, from the number of records identified in the search to the number of studies included in the review, ideally using a flow diagram. | App 3 p3-4 |
|  | 16b | Cite studies that might appear to meet the inclusion criteria, but which were excluded, and explain why they were excluded. | App 3 p3-4 |
| Study characteristics | 17 | Cite each included study and present its characteristics. | Table 2 |
| Risk of bias in studies | 18 | Present assessments of risk of bias for each included study. | Table 2 |
| Results of individual studies | 19 | For all outcomes, present, for each study: (a) summary statistics for each group (where appropriate) and (b) an effect estimate and its precision (e.g. confidence/credible interval), ideally using structured tables or plots. | Table 2 |
| Results of syntheses | 20a | For each synthesis, briefly summarise the characteristics and risk of bias among contributing studies. | Table 2 |
|  | 20b | Present results of all statistical syntheses conducted. If meta-analysis was done, present for each the summary estimate and its precision (e.g. confidence/credible interval) and measures of statistical heterogeneity. If comparing groups, describe the direction of the effect. | Fig 4 |
|  | 20c | Present results of all investigations of possible causes of heterogeneity among study results. | NA |
|  | 20d | Present results of all sensitivity analyses conducted to assess the robustness of the synthesized results. | Fig 4 |
| Reporting biases | 21 | Present assessments of risk of bias due to missing results (arising from reporting biases) for each synthesis assessed. | NA |
| Certainty of evidence | 22 | Present assessments of certainty (or confidence) in the body of evidence for each outcome assessed. | NA |
| **DISCUSSION** | | |  |
| Discussion | 23a | Provide a general interpretation of the results in the context of other evidence. | Discussion p10-11 |
|  | 23b | Discuss any limitations of the evidence included in the review. | Discussion p10-11 |
|  | 23c | Discuss any limitations of the review processes used. | Discussion p10-11 |
|  | 23d | Discuss implications of the results for practice, policy, and future research. | Discussion p10-11 |
| **OTHER INFORMATION** | | |  |
| Registration and protocol | 24a | Provide registration information for the review, including register name and registration number, or state that the review was not registered. | App3 p3-4 |
|  | 24b | Indicate where the review protocol can be accessed, or state that a protocol was not prepared. | App3 p3-4 |
|  | 24c | Describe and explain any amendments to information provided at registration or in the protocol. | NA |
| Support | 25 | Describe sources of financial or non-financial support for the review, and the role of the funders or sponsors in the review. | Methods p9 |
| Competing interests | 26 | Declare any competing interests of review authors. | P14 |
| Availability of data, code and other materials | 27 | Report which of the following are publicly available and where they can be found: template data collection forms; data extracted from included studies; data used for all analyses; analytic code; any other materials used in the review. | P14 |

*From:*  Page MJ, McKenzie JE, Bossuyt PM, Boutron I, Hoffmann TC, Mulrow CD, et al. The PRISMA 2020 statement: an updated guideline for reporting systematic reviews. BMJ 2021;372:n71. doi: 10.1136/bmj.n71

**PRISMA NMA Checklist of Items to Include When Reporting A Systematic Review Involving a Network Meta-analysis** **^67^**

| **Section/Topic** | **Item #** | **Checklist Item** | **Reported on Page #** |
| --- | --- | --- | --- |
| **TITLE** |  |  |  |
| Title | 1 | Identify the report as a systematic review *incorporating a network meta-analysis (or related form of meta-analysis).* | 1 |
|  |  |  |  |
| **ABSTRACT** |  |  |  |
| Structured summary | 2 | Provide a structured summary including, as applicable:  **Background:** main objectives  **Methods:** data sources; study eligibility criteria, participants, and interventions; study appraisal; and *synthesis methods, such as network meta-analysis.*  **Results:** number of studies and participants identified; summary estimates with corresponding confidence/credible intervals; *treatment rankings may also be discussed. Authors may choose to summarize pairwise comparisons against a chosen treatment included in their analyses for brevity.*  **Discussion/Conclusions:** limitations; conclusions and implications of findings.  **Other:** primary source of funding; systematic review registration number with registry name. | 4 |
|  |  |  |  |
| **INTRODUCTION** |  |  |  |
| Rationale | 3 | Describe the rationale for the review in the context of what is already known*, including mention of why a network meta-analysis has been conducted.* | 6 |
| Objectives | 4 | Provide an explicit statement of questions being addressed, with reference to participants, interventions, comparisons, outcomes, and study design (PICOS). | 6 |
|  |  |  |  |
| **METHODS** |  |  |  |
| Protocol and registration | 5 | Indicate whether a review protocol exists and if and where it can be accessed (e.g., Web address); and, if available, provide registration information, including registration number. | Supp. Material page 3 |
| Eligibility criteria | 6 | Specify study characteristics (e.g., PICOS, length of follow-up) and report characteristics (e.g., years considered, language, publication status) used as criteria for eligibility, giving rationale. *Clearly describe eligible treatments included in the treatment network, and note whether any have been clustered or merged into the same node (with justification).* | Table 2 |
| Information sources | 7 | Describe all information sources (e.g., databases with dates of coverage, contact with study authors to identify additional studies) in the search and date last searched. | Supp. Material pages 3-4 |
| Search | 8 | Present full electronic search strategy for at least one database, including any limits used, such that it could be repeated. | Supp. Material pages 3 |
| Study selection | 9 | State the process for selecting studies (i.e., screening, eligibility, included in systematic review, and, if applicable, included in the meta-analysis). | Supp. Material pages 3-4 |
| Data collection process | 10 | Describe method of data extraction from reports (e.g., piloted forms, independently, in duplicate) and any processes for obtaining and confirming data from investigators. | Supp. Material pages 3-4 |
| Data items | 11 | List and define all variables for which data were sought (e.g., PICOS, funding sources) and any assumptions and simplifications made. | Evidence synthesis analysis plan |
| **Geometry of the network** | **S1** | Describe methods used to explore the geometry of the treatment network under study and potential biases related to it. This should include how the evidence base has been graphically summarized for presentation, and what characteristics were compiled and used to describe the evidence base to readers. | Evidence synthesis analysis plan |
| Risk of bias within individual studies | 12 | Describe methods used for assessing risk of bias of individual studies (including specification of whether this was done at the study or outcome level), and how this information is to be used in any data synthesis. | Supp. Material page 4 |
| Summary measures | 13 | State the principal summary measures (e.g., risk ratio, difference in means). *Also describe the use of additional summary measures assessed, such as treatment rankings and surface under the cumulative ranking curve (SUCRA) values, as well as modified approaches used to present summary findings from meta-analyses.* | Pages 7-8 |
| Planned methods of analysis | 14 | Describe the methods of handling data and combining results of studies for each network meta-analysis. This should include, but not be limited to:   - *Handling of multi-arm trials;* - *Selection of variance structure;* - *Selection of prior distributions in Bayesian analyses; and* - *Assessment of model fit.* | Evidence synthesis analysis plan; Appendix 4 |
| **Assessment of Inconsistency** | **S2** | Describe the statistical methods used to evaluate the agreement of direct and indirect evidence in the treatment network(s) studied. Describe efforts taken to address its presence when found. | Appendix 4 |
| Risk of bias across studies | 15 | Specify any assessment of risk of bias that may affect the cumulative evidence (e.g., publication bias, selective reporting within studies). | Page 10  Appendix 4 |
| Additional analyses | 16 | Describe methods of additional analyses if done, indicating which were pre-specified. This may include, but not be limited to, the following:   - Sensitivity or subgroup analyses; - Meta-regression analyses; - *Alternative formulations of the treatment network; and* - *Use of alternative prior distributions for Bayesian analyses (if applicable).* | Appendix 4 & 8 |
| **RESULTS†** |  |  |  |
| Study selection | 17 | Give numbers of studies screened, assessed for eligibility, and included in the review, with reasons for exclusions at each stage, ideally with a flow diagram. | Appendix 4 |
| **Presentation of network structure** | **S3** | Provide a network graph of the included studies to enable visualization of the geometry of the treatment network. | Figure 2 |
| **Summary of network geometry** | **S4** | Provide a brief overview of characteristics of the treatment network. This may include commentary on the abundance of trials and randomized patients for the different interventions and pairwise comparisons in the network, gaps of evidence in the treatment network, and potential biases reflected by the network structure. | Pages 10-11 |
| Study characteristics | 18 | For each study, present characteristics for which data were extracted (e.g., study size, PICOS, follow-up period) and provide the citations. | Tabel 2 |
| Risk of bias within studies | 19 | Present data on risk of bias of each study and, if available, any outcome level assessment. | Appendix 4 |
| Results of individual studies | 20 | For all outcomes considered (benefits or harms), present, for each study: 1) simple summary data for each intervention group, and 2) effect estimates and confidence intervals. *Modified approaches may be needed to deal with information from larger networks.* | Table 2 |
| Synthesis of results | 21 | Present results of each meta-analysis done, including confidence/credible intervals. *In larger networks, authors may focus on comparisons versus a particular comparator (e.g. placebo or standard care), with full findings presented in an appendix. League tables and forest plots may be considered to summarize pairwise comparisons.* If additional summary measures were explored (such as treatment rankings), these should also be presented. | Page 11  Appendix 8 |
| **Exploration for inconsistency** | **S5** | Describe results from investigations of inconsistency. This may include such information as measures of model fit to compare consistency and inconsistency models, *P* values from statistical tests, or summary of inconsistency estimates from different parts of the treatment network. | Appendix 8 |
| Risk of bias across studies | 22 | Present results of any assessment of risk of bias across studies for the evidence base being studied. | Pages 10-11 |
| Results of additional analyses | 23 | Give results of additional analyses, if done (e.g., sensitivity or subgroup analyses, meta-regression analyses*, alternative network geometries studied, alternative choice of prior distributions for Bayesian analyses,* and so forth). | Appendix 8 |
|  |  |  |  |
| **DISCUSSION** |  |  |  |
| Summary of evidence | 24 | Summarize the main findings, including the strength of evidence for each main outcome; consider their relevance to key groups (e.g., healthcare providers, users, and policy-makers). | Page 12 |
| Limitations | 25 | Discuss limitations at study and outcome level (e.g., risk of bias), and at review level (e.g., incomplete retrieval of identified research, reporting bias). *Comment on the validity of the assumptions, such as transitivity and consistency. Comment on any concerns regarding network geometry (e.g., avoidance of certain comparisons).* | Page 13 |
| Conclusions | 26 | Provide a general interpretation of the results in the context of other evidence, and implications for future research. | Page 13 |
|  |  |  |  |
| **FUNDING** |  |  |  |
| Funding | 27 | Describe sources of funding for the systematic review and other support (e.g., supply of data); role of funders for the systematic review. This should also include information regarding whether funding has been received from manufacturers of treatments in the network and/or whether some of the authors are content experts with professional conflicts of interest that could affect use of treatments in the network. | Page 4 |

PICOS = population, intervention, comparators, outcomes, study design.

* Text in italics indicateS wording specific to reporting of network meta-analyses that has been added to guidance from the PRISMA statement.

† Authors may wish to plan for use of appendices to present all relevant information in full detail for items in this section.

# References

1. Mosti G, Mancini S, Bruni S, et al. Adjustable compression wrap devices are cheaper and more effective than inelastic bandages for venous leg ulcer healing. A Multicentric Italian Randomized Clinical Experience. *Phlebology* 2020; **35**(2): 124-33.

2. Gillet JL, Guex JJ, Allaert FA, et al. Clinical superiority of an innovative two-component compression system versus four-component compression system in treatment of active venous leg ulcers: A randomized trial. *Phlebology* 2019; **34**(9): 611-20.

3. O'Meara S, Cullum N, Nelson EA, Dumville JC. Compression for venous leg ulcers. *Cochrane Database of Systematic Reviews* 2012; (11).

4. Harrison MB, Vandenkerkhof EG, Hopman WM, et al. The Canadian Bandaging Trial: Evidence-informed leg ulcer care and the effectiveness of two compression technologies. *BMC Nurs* 2011; **10**: 20.

5. Stather P, Petty C, Langthorne H, et al. A randomised controlled clinical trial comparing the effectiveness of bandaging compared to the JuxtaCures device in the management of people with venous ulceration: Feasibility study. *Phlebology* 2021; **36**(7): 505-14.

6. Lazareth I, Moffatt C, Dissemond J, et al. Efficacy of two compression systems in the management of VLUs: results of a European RCT. *J Wound Care* 2012; **21**(11): 553-4, 6, 8 passim.

7. Sterne JAC, Savović J, Page MJ, et al. RoB 2: a revised tool for assessing risk of bias in randomised trials. *BMJ* 2019; **366**: l4898.

8. Saramago P, Chuang L-H, Soares MO. Network meta-analysis of (individual patient) time to event data alongside (aggregate) count data. *BMC Medical Research Methodology* 2014; **14**(1): 105.

9. Akaike H. Information Theory and an Extension of the Maximum Likelihood Principle. In: Parzen E, Tanabe K, Kitagawa G, eds. Selected Papers of Hirotugu Akaike. New York, NY: Springer New York; 1998: 199-213.

10. Spiegelhalter DJ, Best NG, Carlin BP, Van Der Linde A. Bayesian measures of model complexity and fit. *Journal of the Royal Statistical Society: Series B (Statistical Methodology)* 2002; **64**(4): 583-639.

11. Iglesias C, Nelson EA, Cullum NA, Torgerson DJ. VenUS I: a randomised controlled trial of two types of bandage for treating venous leg ulcers. *Health Technol Assess* 2004; **8**(29): iii, 1-105.

12. Ashby RL, Gabe R, Ali S, et al. VenUS IV (Venous leg Ulcer Study IV) - compression hosiery compared with compression bandaging in the treatment of venous leg ulcers: a randomised controlled trial, mixed-treatment comparison and decision-analytic model. *Health Technol Assess* 2014; **18**(57): 1-293, v-vi.

13. Hernández Alava M, Pudney S, Wailoo A. Estimating the Relationship Between EQ-5D-5L and EQ-5D-3L: Results from a UK Population Study. *Pharmacoeconomics* 2023; **41**(2): 199-207.

14. The National Institute of Health and Care Excellence (NICE). Health technology evaluations: the manual. 2022.

15. Gohel MS, Heatley F, Liu X, et al. Early versus deferred endovenous ablation of superficial venous reflux in patients with venous ulceration: the EVRA RCT. *Health Technol Assess* 2019; **23**(24): 1-96.

16. Hernández-Alava M, Pudney S. Eq5Dmap: A Command for Mapping between EQ-5D-3L and EQ-5D-5L. *The Stata Journal* 2018; **18**(2): 395-415.

17. Pan W. Akaike's information criterion in generalized estimating equations. *Biometrics* 2001; **57**(1): 120-5.

18. Hernández Alava M. PS, Wailoo A. Estimating EQ-5D by Age and Sex for the UK. NICE DSU Report.: NICE DSU Report., 2022.

19. Michaels JA, Campbell WB, King BM, et al. A prospective randomised controlled trial and economic modelling of antimicrobial silver dressings versus non-adherent control dressings for venous leg ulcers: the VULCAN trial. *Health Technol Assess* 2009; **13**(56): 1-114, iii.

20. Office for National Statistics. National life tables. 2022. <https://www.ons.gov.uk/peoplepopulationandcommunity/birthsdeathsandmarriages/lifeexpectancies/datasets/nationallifetablesunitedkingdomreferencetables> (accessed Aug 2024.

21. Joint Formulary Committee (JFC). British National Formulary. 2024. <https://bnf.nice.org.uk/>.

22. National Health Service. National Cost Collection for the NHS 2023/2024. 2024. <https://www.england.nhs.uk/costing-in-the-nhs/national-cost-collection/>.

23. Personal Social Service Research Unit. Unit costs of health and social care 2023. 2023. <https://www.pssru.ac.uk/project-pages/unit-costs/> (accessed date unknown].

24. Economic - narrow - MEDLINE, Embase. CADTH Search Filters Database. <https://searchfilters.cadth.ca/link/20> (accessed 8th August 2024 2024).

25. Brain D, Tulleners R, Lee X, Cheng Q, Graves N, Pacella R. Cost-effectiveness analysis of an innovative model of care for chronic wounds patients. *PLoS ONE* 2019; **14(3) (no pagination)**(e0212366).

26. Taylor RR, Sladkevicius E, Guest JF. Modelling the cost-effectiveness of electric stimulation therapy in non-healing venous leg ulcers. *J Wound Care* 2011; **20**(10): 464, 6, 8-72.

27. Panca M, Cutting K, Guest JF. Clinical and cost-effectiveness of absorbent dressings in the treatment of highly exuding VLUs. *J Wound Care* 2013; **22**(3): 109-10, 12.

28. Jemec GB, Kerihuel JC, Ousey K, Lauemoller SL, Leaper DJ. Cost-effective use of silver dressings for the treatment of hard-to-heal chronic venous leg ulcers. *PLoS ONE* 2014; **9**(6): e100582.

29. Carter MJ, Waycaster C, Schaum K, Gilligan AM. Cost-effectiveness of three adjunct cellular/tissue-derived products used in the management of chronic venous leg ulcers. *Value Health* 2014; **17**(8): 801-13.

30. Romanelli M, Gilligan AM, Waycaster CR, Dini V. Difficult-to-heal wounds of mixed arterial/venous and venous etiology: a cost-effectiveness analysis of extracellular matrix. *ClinicoEcon* 2016; **8**: 153-61.

31. Augustin M, Herberger K, Kroeger K, Muenter KC, Goepel L, Rychlik R. Cost-effectiveness of treating vascular leg ulcers with UrgoStart( R) and UrgoCell( R) Contact. *Int Wound J* 2016; **13**(1): 82-7.

32. Nherera LM, Woodmansey E, Trueman P, Gibbons GW. Estimating the Clinical Outcomes and Cost Differences Between Standard Care With and Without Cadexomer Iodine in the Management of Chronic Venous Leg Ulcers Using a Markov Model. *Ostomy Wound Manage* 2016; **62**(6): 26-40.

33. Guest JF, Weidlich D, Singh H, et al. Cost-effectiveness of using adjunctive porcine small intestine submucosa tri-layer matrix compared with standard care in managing diabetic foot ulcers in the US. *J Wound Care* 2017; **26**(Sup1): S12-S24.

34. Epstein D, Gohel M, Heatley F, Davies AH. Cost-effectiveness of treatments for superficial venous reflux in patients with chronic venous ulceration. *BJS open* 2018; **2**(4): 203-12.

35. Gueltzow M, Khalilpour P, Kolbe K, Zoellner Y. Budget impact of antimicrobial wound dressings in the treatment of venous leg ulcers in the German outpatient care sector: a budget impact analysis. *J Mark Access Health Policy* 2018; **6**(1): 1527654.

36. Guest JF, Rana K, Singh H, Vowden P. Cost-effectiveness of using a collagen-containing dressing plus compression therapy in non-healing venous leg ulcers. *J Wound Care* 2018; **27**(2): 68-78.

37. Walzer S, Droschel D, Vollmer L, Atkin L, Ousey K. A cost-effectiveness analysis of a hydration response technology dressing in the treatment of venous leg ulcers in the UK. *J Wound Care* 2018; **27**(3): 166-72.

38. Cheng Q, Gibb M, Graves N, Finlayson K, Pacella RE. Cost-effectiveness analysis of guideline-based optimal care for venous leg ulcers in Australia. *BMC Health Serv Res* 2018; **18**(1): 421.

39. Ontario HQ. Compression Stockings for the Prevention of Venous Leg Ulcer Recurrence: A Health Technology Assessment. *Ont Health Technol Assess Ser* 2019; **19**(2): 1-86.

40. Djalalov S, Sehatzadeh S, Keast DH, Wong WW. Economic evaluation of compression stockings for the prevention of venous leg ulcer recurrence in Ontario. *J Wound Care* 2020; **29**(3): 141-51.

41. Kirsner RS, Delhougne G, Searle RJ. A Cost-Effectiveness Analysis Comparing Single-use and Traditional Negative Pressure Wound Therapy to Treat Chronic Venous and Diabetic Foot Ulcers. *Wound Manag Prev* 2020; **66**(3): 30-6.

42. Rognoni C, Lugli M, Maleti O, Tarricone R. Venous stenting for patients with outflow obstruction and leg ulcers: cost-effectiveness and budget impact analyses. *J* 2020; **9**(10): 705-20.

43. Guest JF, Atkin L, Aitkins C. Potential cost-effectiveness of using adjunctive dehydrated human amnion/chorion membrane allograft in the management of non-healing diabetic foot ulcers in the United Kingdom. *Int Wound J* 2021; **18**(6): 889-901.

44. Ontario HQ. Skin Substitutes for Adults With Diabetic Foot Ulcers and Venous Leg Ulcers: A Health Technology Assessment. *Ont Health Technol Assess Ser* 2021; **21**(7): 1-165.

45. Guest JF, Staines K, Murphy N. Cost-effectiveness of using intermittent pneumatic compression to manage hard-to-heal venous leg ulcers in the UK. *J Wound Care* 2021; **30**(7): 544-52.

46. Velickovic VM, Szilcz M, Milosevic Z, Godfrey T, Siebert U. Cost-effectiveness analysis of superabsorbent wound dressings in patients with moderate-to-highly exuding leg ulcers in Germany. *Int Wound J* 2022; **19**(2): 447-59.

47. Velickovic VM, Prieto PA, Krga M, Jorge AM. Superabsorbent wound dressings versus foams dressings for the management of moderate-to-highly exuding venous leg ulcers in French settings: An early stage model-based economic evaluation. *J Tissue Viability* 2022; **31**(3): 523-30.

48. Guest JF, Deanesi V, Segalla A. Cost-effectiveness of Debrichem in managing hard-to-heal venous leg ulcers in the UK. *J Wound Care* 2022; **31**(6): 480-91.

49. Zheng H, Magee GA, Tan TW, Armstrong DG, Padula WV. Cost-effectiveness of Compression Therapy With Early Endovenous Ablation in Venous Ulceration for a Medicare Population. *JAMA netw* 2022; **5**(12): e2248152.

50. Velickovic VM, Lembelembe JP, Cegri F, et al. Superabsorbent Wound Dressing for Management of Patients With Moderate-to-Highly Exuding Chronic Leg Ulcers: An Early Stage Model-Based Benefit-Harm Assessment. *Int* 2023; **22**(2): 345-52.

51. Guest JF, Fuller GW. Relative cost-effectiveness of three compression bandages in treating newly diagnosed venous leg ulcers in the UK. *J Wound Care* 2023; **32**(3): 146-58.

52. Cooper DM, Bojke C, Ghosh P. Cost-Effectiveness of PHMB & betaine wound bed preparation compared with standard care in venous leg ulcers: A cost-utility analysis in the United Kingdom. *J Tissue Viability* 2023; **32**(2): 262-9.

53. Guest JF, Fuller GW. Cost-effectiveness of two reduced pressure compression systems in treating newly diagnosed venous leg ulcers. *J Wound Care* 2023; **32**(6): 348-58.

54. Guest JF, Apelqvist J. Cost-effectiveness of ChloraSolv in treating hard-to-heal venous leg ulcers. *J Wound Care* 2024; **33**(1): 4-13.

55. Cacua Sanchez MT, Botero AM, Moreno-Mattar O. Cost-effectiveness analysis of intralesional and perilesional recombinant human epidermal growth factor vs hydrocolloid therapy in venous ulcer treatment in the Colombian context. *J Vasc Surg Venous Lymphat Disord* 2024; **12**(2): 101745.

56. Salenius JE, Suntila M, Ahti T, et al. Long-term Mortality among Patients with Chronic Ulcers. *Acta Dermato-Venereologica* 2021; **101**(5): adv00455.

57. Kreft D, Keiler J, Grambow E, Kischkel S, Wree A, Doblhammer G. Prevalence and Mortality of Venous Leg Diseases of the Deep Veins: An Observational Cohort Study Based on German Health Claims Data. *Angiology* 2020; **71**(5): 452-64.

58. Pham B, Harrison MB, Chen MH, Carley ME, for the Canadian Bandaging Trial G. Cost-effectiveness of compression technologies for evidence-informed leg ulcer care: results from the Canadian Bandaging Trial. *BMC Health Services Research* 2012; **12**(1): 346.

59. Cheng Q, Kularatna S, Lee XJ, Graves N, Pacella RE. Comparison of EQ-5D-5L and SPVU-5D for measuring quality of life in patients with venous leg ulcers in an Australian setting. *Qual Life Res* 2019; **28**(7): 1903-11.

60. Barnsbee L, Cheng Q, Tulleners R, Lee X, Brain D, Pacella R. Measuring costs and quality of life for venous leg ulcers. *Int Wound J* 2019; **16**(1): 112-21.

61. Balieva F, Kupfer J, Lien L, et al. The burden of common skin diseases assessed with the EQ5D™: a European multicentre study in 13 countries. *Br J Dermatol* 2017; **176**(5): 1170-8.

62. Yang Y, Brazier J, Longworth L. EQ-5D in skin conditions: an assessment of validity and responsiveness. *Eur J Health Econ* 2015; **16**(9): 927-39.

63. The Core Model. Network Meta‐Analysis for Decision Making; 2018: 19-58.

64. Spruance SL, Reid JE, Grace M, Samore M. Hazard ratio in clinical trials. *Antimicrob Agents Chemother* 2004; **48**(8): 2787-92.

65. Papakonstantinou T, Nikolakopoulou A, Higgins JPT, Egger M, Salanti G. CINeMA: Software for semiautomated assessment of the confidence in the results of network meta-analysis. *Campbell Syst Rev* 2020; **16**(1): e1080.

66. Nikolakopoulou A, Higgins JPT, Papakonstantinou T, et al. CINeMA: An approach for assessing confidence in the results of a network meta-analysis. *PLoS Med* 2020; **17**(4): e1003082.

67. Hutton B, Salanti G, Caldwell DM, et al. The PRISMA Extension Statement for Reporting of Systematic Reviews Incorporating Network Meta-analyses of Health Care Interventions: Checklist and Explanations. *Annals of Internal Medicine* 2015; **162**(11): 777-84.
