# Supplementary material for: Compression systems for venous leg ulcers: a network meta-analysis and cost-effectiveness analysis
Source: eClinicalMedicine. 2026 Jul 14;97:104065. doi: 10.1016/j.eclinm.2026.104065 (PMC13382440; doi:10.1016/j.eclinm.2026.104065)
Supplement: VenUS 6 investigators [file mmc2.docx]

**VenUS 6 investigators:**

- Ross Atkinson
- Una Adderley
- Ian Chetter
- Nicky Cullum
- Tom Davill
- Jane Griffiths
- Catherine Hewitt
- Charlotte Hirst
- Katherine Jones
- Maartje Kletter
- Julie Mullings
- Gareth Roberts
- Brigid Smart
- Philip Stather
- Nikki Stubbs
- Jude Watson
- Sabeen Zahra
